# Profiling of Yeast *Saccharomyces cerevisiae* Mitochondrial AMPylome Reveals a Regulation of OXPHOS Coupling through ATP Synthase Subunit Delta

**DOI:** 10.1021/acs.jproteome.5c01273

**Published:** 2026-07-28

**Authors:** Chiranjit Panja, Aneta Wiesyk, Suchismita Masanta, Katarzyna Niedzwiecka, Emilia Baranowska, Dominik Cysewski, Marta Sipko, Anna Anielska-Mazur, Agata Malinowska, Roza Kucharczyk

**Affiliations:** Institute of Biochemistry and Biophysics, Polish Academy of Sciences, Pawinskiego 5A, 02-106 Warsaw, Poland

**Keywords:** AMPylation, yeast, mitochondria, Fmp40, ATP synthase

## Abstract

Protein adenylation (AMPylation) is a post-translational modification in which an adenosine monophosphate (AMP) group is covalently attached to target proteins by AMPylases using ATP as a donor. In metazoans, two conserved AMPylase families are known: FIC-domain proteins and SelO. The yeast *Saccharomyces cerevisiae* lacks a FIC-domain enzyme; its only known AMPylase is the mitochondrial SelO homologue, Fmp40, involved in redox signaling. We conducted the first comprehensive screen for AMPylated proteins in the mitochondrial proteome of *S. cerevisiae* analyzing both wild-type and *fmp40*Δ cells using quantitative mass spectrometry. We identified 124 AMPylated mitochondrial proteins in wild-type and 41 in *fmp40*Δ mitochondria, suggesting the existence of additional AMPylase(s) in yeast. Among the modified targets, seven ATP synthase subunits were AMPylated, many at sites also phosphorylated, underscoring complex PTM regulation of the enzyme. We demonstrated that substitutions of one such residue, serine 29 in the δ subunit (Atp16), to alanine or glutamic acid, altered ATP synthase activity and oxidative phosphorylation coupling under both fermentative and respiratory conditions. This regulation is crucial for maintaining mitochondrial membrane potential. Our study provides the first catalog of AMPylated mitochondrial proteins in yeast, establishing a foundation for future studies on mitochondrial AMPylation.

## Graphical abstract

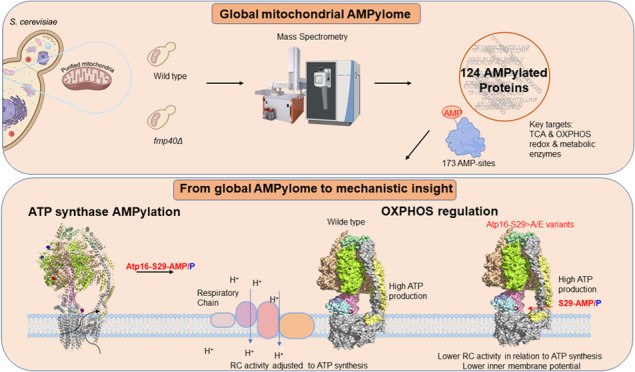

## Introduction

Mitochondria perform multiple essential functions within the cell, the most prominent of which is ATP production via oxidative phosphorylation (OXPHOS), a process driven by multisubunit enzymes of the respiratory chain and ATP synthase organized into supercomplexes within the inner mitochondrial membrane.^1^ During this process, electrons derived from carbohydrates and fatty acids are transferred to oxygen through the respiratory chain, which concomitantly pumps protons from the mitochondrial matrix into the intermembrane space, thereby generating a proton gradient across the inner mitochondrial membrane. The proton gradient is then utilized by ATP synthase to phosphorylate ADP to ATP, contributing to the proper polarization of the inner mitochondrial membrane (ΔΨ).^2^ During ATP synthesis, protons flow from the intermembrane space into the matrix through the membrane-embedded channel of ATP synthase, formed by the *c*/Atp9-ring and subunit *a* (Atp6). Proton translocation drives rotation of the *c*-ring together with the three central stalk subunits of the catalytic domain. Rotation of this rotor induces conformational changes within the catalytic α/Atp1_3_β/Atp2_3_ hexamer, promoting ADP and inorganic phosphate binding, ATP synthesis, and subsequent ATP release. The process is tightly coupled: three ATP molecules are produced per full rotation of the *c*-ring, and the number of protons translocated during one cycle corresponds to the number of *c* subunits within the ring.^3^ The proton flux through ATP synthase is continuously compensated by proton pumping to the intermembrane space via the respiratory chain, and the precise mechanisms regulating the respiratory chain coupling to ATP synthase activity are not fully understood.^4–6^

Beyond ATP production, mitochondria play a central role in numerous metabolic pathways, including the synthesis of amino acids, lipids, heme, and iron–sulfur clusters. They also serve as signaling hubs that can trigger programmed cell death.^7^ In addition, mitochondria contain their own DNA (mtDNA), which encodes several subunits of the OXPHOS machinery. Therefore, mitochondrial biogenesis—which adjusts to the cell’s energy demands—requires coordinated gene expression between the nuclear and mitochondrial genomes, as well as the import of nuclear-encoded mitochondrial proteins from the cytoplasm into the organelle.^8^ The activity of the OXPHOS system is accompanied by the continuous production of reactive oxygen species (ROS), which play important roles in cellular signaling.^9^ All these processes must be tightly regulated to allow mitochondria to adapt to changes in energy demand, cellular metabolism, and environmental conditions.^10^ Mitochondrial and cellular functions are regulated at multiple levels, including gene expression—coordinated between the nuclear and mitochondrial genomes—mRNA synthesis and turnover, protein synthesis and degradation, and protein activity through posttranslational modifications (PTMs). One such PTM is phosphorylation, where a phosphoryl group (PO_3_^–^) is added to hydroxyl-containing amino acids such as serine, threonine, and tyrosine by protein kinases.^11^ Studies by various research groups investigating the regulation of the mitochondrial proteome by phosphorylation have revealed that approximately 67% of mitochondrial proteins are phosphorylated, with an average of 6.76 phosphorylation sites per protein.^12–15^ The biological significance of some of these modifications has been described in the literature, including those found in the Atp2 and Atp20 subunits of ATP synthase, as well as in the Qcr2 subunit of complex III.^12,16–18^

The same amino acid residues that are phosphorylated can also undergo another modification: the covalent attachment of adenosine monophosphate (AMP), known as AMPylation or adenylation. This modification was first discovered in 1967 as a mechanism regulating the activity of bacterial glutamine synthetase.^19–21^ AMPylation was first identified in eukaryotes in the context of bacterial infection, when Yarbrough et al. reported the AMPylation of host small GTPases by the bacterial virulence factor VopS from*Vibrio parahemolyticus*. This modification prevents GTPases from interacting with their downstream binding partners, causing actin cytoskeleton collapse and ultimately leading to host cell death.^22^ Bacterial AMP transferases have been classified into two main categories: filamentation induced by cyclic AMP (FIC) and adenylyl transferase (AT) domain-containing enzymes.^20,23^ In humans, there is one FIC domain-containing AMPylase, FicD (also known as HYPE, encoded by the *FICD* gene), which is localized in the endoplasmic reticulum and modulates the unfolded protein response by AMPylating the HSP70 family chaperone BiP.^24,25^ In recent years, the role of FicD-mediated AMPylation has been linked to diabetes^26,27^ and other clinical manifestations, including neurological disorders like Parkinson’s disease, hereditary spastic paraplegia, and polyQ-mediated cellular toxicity that can affect a spectrum of neurological disorders mediated by polyQ protein toxicity.^26,28^ This function of Fic AMPylases is conserved across other organisms.^29,30^ In 2018, a novel class of AMPylases was discovered: proteins from the Selenoprotein O (SelO) family, which were previously classified as pseudokinases.^31,32^ Both mammalian and *S. cerevisiae* SelO (Fmp40) are localized in the mitochondria, regulated by their redox state, and are active when reduced.^32,33^ The biological role of SelO is to maintain redox homeostasis in this organelle by regulating global protein glutathionylation.^32^ In yeast, Fmp40 levels are induced upon respiration and exposure to hydrogen peroxide. Mitochondrial redoxins Prx1, Trx3, and Grx2 are AMPylated by Fmp40 *in vitro*, and physical interactions between Fmp40 and Trx3, as well as Prx1, have been demonstrated *in vivo*.^34^ Fmp40 regulates the redox cycles of Prx1 and Trx3, with one mechanism being the AMPylation of Trx3′s T66 residue, which is crucial to maintain proper Trx3 levels and maturation during mitochondrial matrix import.^34^ To date, Fmp40 is the only AMPylase identified in yeast, as this organism lacks a FIC domain-containing protein.^25^

To understand the biological function of FicD AMPylases in metazoans, many groups have employed various approaches to identify the substrates of these enzymes. *In vitro* chemo-proteomic screening with an ATP analogue, Yn-6-ATP, and chemoenzymatic tagging of AMP by biotin, followed by substrate validation for HYPE-mediated AMPylation in mammalian HEK293 cell lysates, led to the identification of 25 AMPylated proteins.^35^ Using N6-propargyl-ATP (N6pA) and chemical coupling of biotin-(PEG)­3-azide to add a biotin handle to the ATP-bound propargyl group, HSP70 proteins and translation elongation factors were purified from *C. elegans* cell lysates and mouse embryonic fibroblast cell lysates, among the 108 AMPylated proteins identified.^29,36^ The cosubstrate-mediated covalent capture strategy, based on the covalent reaction of strategically placed cysteines in the active center of AMPylase with synthetic thiol-reactive nucleotide derivatives, allows for the identification of its target proteins by generating a stable covalent complex between the AMPylase and the target protein.^37^ The development of the cell-permeable adenosine pro-nucleotide probe (pro-N6pA), which is metabolically converted to N6pATP, enables cellular AMPylation reactions followed by click-chemistry, allowing the screening of AMPylated proteins directly from living cells.^38^ Using this approach, a total of 162 AMPylated proteins were identified across five different cell lines. Affinity purification with antibodies against adenosine-phosphate and anti-Tyr-AMP was performed to isolate AMPylated proteins from extracts of human chronic lymphocytic leukemia FicD wild-type and FicD knockout cell lines.^39^ A total of 213 proteins were identified from wild-type cell extracts, whereas only 23 were pulled down from FicD-deficient cells. SelO substrates were identified using *in vitro* approaches in*Escherichia coli*, human mitochondria, and *in vivo* in *Arabidopsis thaliana*.^32,40,41^ To screen for human SelO substrates, recombinant wild-type SelO or inactive SelO were incubated with mitochondria from YUMM3.3 subcutaneous tumors and a biotinylated ATP analog, bio-17-ATP. The list of proteins enriched with the wild-type enzyme versus the inactive enzyme includes 491 proteins. In another approach to identify SelO-dependent AMPylated proteins using a nucleotide-binding protein hint, 124 proteins were identified, including glutamate dehydrogenase and pyruvate dehydrogenase, whose AMPylation regulates metabolic flux.^42^ However, the complete range of SelO targets in other model organisms remains to be determined.

This study presents the first comprehensive analysis of the AMPylated proteins in mitochondria isolated from *Saccharomyces cerevisiae*. By employing both global and affinity purification approaches targeting mitochondrial ATP synthase, Trx3-myc redoxin, and Fmp40-myc AMPylase, we identified 124 AMPylated proteins and 173 distinct AMPylation sites within a high-confidence mitochondrial proteome. AMPylation of ATP synthase subunits was detected following ATP synthase complex pull-down from native mitochondria or after its liberation from the mitochondrial membrane with digitonin. In parallel, we analyzed mitochondrial protein phosphorylation, revealing novel phosphorylation sites that had not previously been found in the yeast mitochondrial phosphoproteome. Newly identified phosphoproteins include OXPHOS complex subunits (Qcr10, Cox5a, Cox9, Cox13, Cox2), the mitochondrial import machinery component Tim23, and mitochondrial thioredoxin Trx3, suggesting a greater extent of mitochondrial protein phosphorylation than previously recognized. Importantly, we demonstrate the critical role of the serine 29 residue in Atp16 ATP synthase subunit—which can be modified by both AMPylation and phosphorylation—in coupling ATP synthase to the respiratory chain and maintaining the mitochondrial inner membrane potential during growth on both fermentable and nonfermentable carbon sources.

## Experimental Procedures

### Yeast Strains, Media, and Growth Conditions

The MR6 strain (*MATa ade2-1 his3-1*,*15 leu2-3*,*112 trp1-1 ura3-1 arg8::HIS3*) used in the study is a derivative of W303-1B strain background (Table 1).^43^ The strain expressing the *ATP6-HA-6xHIS* gene was a gift from Prof. Alexander Tzagoloff (Columbia University, NY, USA^44^). The strain expressing the *TRX3-MYC* gene was a kind gift from Prof. Chris Grant (University of Manchester, UK,^45^) and was used to receive the RKY206 strain. Strains were grown in rich YPGA medium (1% Bacto yeast extract, 1% Bacto peptone, 2% glucose, 40 mg/L adenine), YPGlyA medium (1% Bacto yeast extract, 1% Bacto peptone, 2% glycerol, 40 mg/L adenine), or SC selective medium (6.7 g/L YNB w/o amino acids, 2% glucose or glycerol, 40 mg/L adenine, appropriate amino-acids drop-out powder (Sunrise)) at 28 or 36 °C with shaking at 180 rpm.

**Table 1:** Genotypes and Sources of Yeast Strains

Strain	Nuclear genotype	mtDNA	Source
MR6	*MATa ade2-1 his3-11*,*15 trp1-1 leu2-3*,*112 ura3-1 CAN1 arg8::HIS3*	ρ^+^	^43^
RKY120	*MATa ade2-1 his3-11*,*15 trp1-1 leu2-3*,*112 ura3-1 CAN1 arg8::HIS3 fmp40::KANMX4*	ρ^+^	^32^
MR6-ATP6-HAHis	*MATa ade2-1 his3-11*,*15 trp1-1 leu2-3*,*112 ura3-1 CAN1 arg8::HIS3*	ρ^+^ *ATP6-HA-6xHis*	^44^
RKY206	*MATα leu2-3*,*112 trp1-1 ura3-1 ade2-1 his3-11*,*15 CAN1 arg8::hisG TRX3-MYC::kanMX6*	ρ^+^	^34^
RKY193–2A	*MATa leu2-3*,*112 trp1-1 ura3-1 ade2-1 his3-11*,*15 CAN1 arg8::hisG fmp40::kanMX4 TRX3-MYC::kanMX6*	ρ^+^	^34^
RKY324	*MATα leu2-3*,*112 trp1-1 ura3-1 ade2-1 his3-11*,*15 CAN1 arg8::hisG atp16::kanMX4/pFL38-ATP16*	ρ^+^	This study
RKY362	*MATα leu2-3*,*112 trp1-1 ura3-1 ade2-1 his3-11*,*15 CAN1 arg8::hisG atp16::kanMX4/pFL38-ATP16-S29>A*	ρ^+^	This study
RKY363	*MATα leu2-3*,*112 trp1-1 ura3-1 ade2-1 his3-11*,*15 CAN1 arg8::hisG atp16::kanMX4/pFL38-ATP16-S29>E*	ρ^+^	This study
atp16Δ	*MATa ade2-1 his3-11*,*15 trp1-1 leu2-3*,*112 ura3-1 CAN1 arg8::HIS3 atp16::kanMX4*	ρ^0^	This study

The Atp16, Atp16-S29>A, and Atp16-S29>E were produced from *ATP16* gene variants cloned into the centromeric pFL38 plasmid (the pFL38-ATP16 was provided by Dr. Michaela Carraro^46^). The site-directed mutagenesis was performed according to the protocol of Q5 Site-Directed Mutagenesis Kit (*NEBiolabs*) using this plasmid as a template with the pairs of primers: ATP16-S29>A 5′GTTCATATGCAGAAGCTGCTGCCGCA**GCA**TCAGGTT TGAAGTTACAATTTGC3′ or ATP16-S29>E 5′GTTCATATGCAGAAGCTGCTGCCGCA **GAA**TCAGGTTTGAAGTTACAATTTGC3′ and ATP16-S29short 5′GCTTAGCGACGAAATTCAATG3′. The correct sequence of *ATP16* gene variants in resulting plasmids, pRK98 and pRK99, respectively, was verified by sequencing. Due to the massive loss of mtDNA in *atp16*Δ cells, the plasmids were introduced into the wild-type MR6 strain before deletion of the wild-type *ATP16* gene sequence. Such strains were transformed with an *atp16::KANMX4* deletion cassette, amplified with ATP16-Up and ATP16-Low primers (5′TGCCCAGCCAATCAAAGCGC3′, 5′CGATGATCTGCAACTAGGGC3′, respectively) using the total DNA from *atp16::KANMX4* knockout strain obtained from Open Biosystems. Transformants were selected on the YPGA + Geneticin (200 μg/mL) plates. The correct integration of the cassette into the genomic *ATP16* locus in RKY324, RKY362, and RKY363 (Table 1), not into the plasmid-born copies, was verified by PCR with primers ATP16-low and Kan-dln (5′GATTTTGATGACGAGCGTAATG). The primer ATP16-low sequence is absent in the plasmid pFL38-ATP16.

### Isolation of Pure Mitochondria for Mass Spectrometry (MS) Analysis

Mitochondria were isolated from cells grown in YPGlyA at 28 °C by the previously described enzymatic method, , with minor modifications.^47^ Briefly, cells grown to an OD_600_of 4 were centrifuged, washed with cold water, and incubated for 10 min in the SH buffer (0.1 M Tris, pH 9.3, 0.5 M 2-β-mercaptoethanol) at 32 °C. After washing twice with the KCl buffer (10 mM Tris pH 7.0, 0.5 M KCl) cells were digested by Zymolyase T20 (ref 07663-91, Nacalai Tresque, Japan) at 8 mg/1 g of dry weight calculated from the equation (number of liters x OD of the culture × 0.28) in the 10 mL/1 g of dry weight Digestion buffer (1.35 M sorbitol, 1 mM EGTA, 10 mM citric acid, 30 mM disodium phosphate) for 30 min at 32 °C. Spheroplasts were washed twice with the Washing buffer (10 mM Tris-maleate, pH 6.8, 0.75 M sorbitol, 0.4 M mannitol, 0.1% BSA), suspended in 40 mL of Homogenization buffer (10 mM Tris-maleate, pH 6.8, 0.6 M mannitol, 2 mM EGTA, 0.2% BSA), and homogenized 3 × 5 s. in the Mixer blender in the 37 mL inox cup (WARING, #W67902, #W87278S). After centrifugation of the cell debris at 1076*g* for 8 min, the homogenate was centrifuged at 11952*g* for 10 min at 4 °C (Sorval RC 5BPlus, SS-34 rotor). Mitochondria were washed once with the Recuperation buffer (10 mM Tris-maleate, pH 6.8, 0.6 M mannitol, 2 mM EGTA) and suspended in 9 mL of 15% Percoll in the Recuperation buffer. The mitochondria were placed on the 23–40% Percoll-Recuperation buffer gradient and purified by ultracentrifugation in Sorvall WX Ultra Series Centrifuge (Thermo Fisher Scientific) at conditions: 31176*g* (28000 rpm), 14 min, 4 °C, acceleration 2, deceleration 1, T-8100 rotor. The pure mitochondrial fraction was picked from 23–40% phase interface, washed twice in the Recuperation buffer and centrifuged in the Eppendorf 5804R centrifuge at 15557*g*, 20 min, 4 °C, F34-6-38 rotor. Mitochondria were suspended in 250 μL of sonication buffer (100 mM Tris-HCl, pH 7.4; 6 M guanidine hydrochloride, inhibitors of proteases cOmplete Mini (Roche, 11836153001), inhibitors of phosphatases Phosphatase Inhibitor Cocktail I Liquid (Thermo Fisher Scientific, J63907) and sonicated in Bioruptor XL Diagenode 30 times 30 s at “high” with 30 s intervals on ice. To preserve AMPylation, proteins were precipitated using cold acetone (1 mL precooled acetone was added to the sample and incubated for 1 h at −20 °C, then harvested using an OHAUS FC5515R centrifuge at 9500*g*, 10 min at 4 °C). The protein pellets were dried at 37 °C for 30 min and analyzed by mass spectrometry. The experiment was repeated ten times for each strain, the wild-type, and *fmp40*Δ.

### Affinity Purification of Proteins from Mitochondria

To pull down the ATP synthase monomer, the strain expressing *ATP6-HA-6xHIS* from the mitochondrial genome was used.^48^ The whole ATP synthase complexes were purified from 5 mg of mitochondria by Ni-NTA agarose beads as described in the reference.^49^ The 2 μg of Ni-NTA bead eluate was loaded onto the 15% SDS-PAGE gel. Then the gel was stained with Silver Stain Kit according to the manufacturer’s protocol (Pierce silver stain kit, Thermo Fisher Scientific), to visualize the proteins before MS analysis. In parallel, the proteins after electrophoretic separation were transferred to a nitrocellulose membrane and processed by Western blotting with antibodies against Atp2, Atp3, Atp4, Atp5, Atp16 (a kind gift from Dr. Marie-France Giraud, Bordeaux), anti-AMP-7C11 (kindly provided by Prof. Aymelt Itzen,^50^), and anti-Tyr-AMP (ref ABS184, Merck). The experiment was performed five times.

The ATP synthase dimers and monomers were extracted from 1 mg of the mitochondria, divided into 5 tubes of 2 mL in extraction buffer (30 mM HEPES pH 6.8, 150 mM potassium acetate, 12% glycerol, 2 mM 6-aminocaproic acid, 1 mM EGTA, 1.5% digitonin (Sigma), inhibitors of proteases cOmplete Mini, by incubation on ice for 26 min. The samples were centrifuged using an OHAUS FC5515R centrifuge at 21950*g*, 4 °C, for 30 min, supplemented with 4.5 μL of loading dye (5% Serva Blue G-250, 750 mM 6-aminocaproic acid), and run on NativePAGE 3–12% Bis-Tris Gels (Thermo Fisher Scientific). Dimers and monomers were cut off from the gel and analyzed by MS. The experiment was performed in triplicate for each strain: the wild-type and *fmp40*Δ.

For Trx3-Myc and Fmp40-Myc purification, the RKY206 (Table 1) or wild-type and RKY120 transformed with pUG35-based plasmid pSP2 (encoding Fmp40-Myc C-terminal fusion) were used. One mg of mitochondria isolated from cells grown on rich or minimal selective lacking uracil, glycerol-containing media, was centrifuged using OHAUS FC5515R centrifuge at 9500*g* for 10 min and suspended in 400 μL of the solubilization buffer (20 mM Tris/HCl pH 7.4, 0.1 mM EDTA, 50 mM NaCl, 10% [vol/vol] glycerol, 1% [wt/vol] digitonin, 2 mM PMSF, inhibitors of proteases cOmplete Mini) and incubated for 30 min on ice. After centrifugation as above, the supernatant was collected and incubated with 200 μL of Pierce Anti-c-Myc Magnetic beads (Thermo Fisher Scientific). The bound proteins were analyzed by MS. The above experiments were done in duplicate for each strain, the wild-type and *fmp40*Δ.

### 2nd Dimension Electrophoresis of ATP Synthase Complexes and Western Blotting

For second-dimension electrophoresis, the dimers and the monomers were cut off from the first BN-PAGE gel and incubated for 10 min in the SDS-PAGE electrophoresis buffer supplemented with β-mercaptoethanol (50 μL per 10 mL of buffer), placed into the lanes of the Novex Tris-Glycine Mini Protein 16% gel (Thermo Fisher Scientific). Proteins were transferred to the cellulose membrane with the iBlot2 Gel Transfer Device (Thermo Fisher Scientific) and followed by Western blotting with polyclonal antibodies against Atp2, Atp3, Atp4, Atp5, Atp16, anti-Tyr-AMP (ref ABS184, Merck), and anti-Thr-AMP (ref 09-890, Merck). All antibodies were generated in a rabbit. The experiment was performed three times.

### Mass Spectrometry Analysis

The tryptic digested peptides were analyzed by LC/MS system at the IBB PAS Mass Spectrometry Facility unit using Evosep One (Evosep Biosystems, Odense, Denmark) coupled to an Orbitrap Exploris 480 mass spectrometer (Thermo Fisher Scientific, Bremen, Germany) via Flex nanoESI ion source (2) or by a nanoACQUITY UPLC system (Waters, Milford, MA) coupled to either Orbitrap Velos or Q Exactive mass spectrometer (Thermo Fisher Scientific, Bremen, Germany). Data were collected in a data-dependent manner in positive mode. Part of data was also preprocessed with Mascot Distiller (version 2.8, Matrixscience) and searched with Mascot search engine (version 2.8, Matrixscience) against the SGD database (version 20210423; 6624 protein sequences), with offline mass recalibration (MScan software, version 3.0, https://proteom.ibb.waw.pl/mscan/) based on results of preliminary Mascot searches, typical resulting parent mass window of 5 ppm and fragment mass window of 0.01 Da. The rest of the search parameters were similar to the ones used in Proteome Discoverer searches described below, which formed the main body of analysis. Results were validated using a target/decoy strategy, with a threshold q value of 0.01. Proteome Discoverer (version 2.4.1.15, Thermo) software was used to preprocess obtained files, perform peptide/protein identification with the Sequest search engine, and validate the results and localize posttranslational modifications. First, data was searched against the SGD database (version 20210423; 6624 of protein entries searched in the database using Proteome Discoverer) supplemented with the database of popular contaminants (CRAP), with the following parameters—enzyme: semiTrypsin, missed cleavages: 2, precursor mass: 10 ppm, fragment mass: 0.02 Da, variable modifications: Oxidation (M), Phosphoadenosine (HKSTY), Phosphorylation (STY). Semitryptic specificity was used as a search parameter to increase the sensitivity of PTM identification, taking into account possible endogenous proteolytic cleavage and incomplete trypsin digestion, while retaining a reasonable size of search space and reliable FDR level.^51,52^ Fixed modification depended on the sample preparation method and was defined either as Carbamidomethyl (C), Methylthio (C), or none. Search results were later validated with Percolator, with target FDR for PSM, peptide, and protein level equal to 0.01.^53^ The data were then processed using the IMP-ptmRS module, a modification site localization tool, which calculates site probabilities, subsequently used to filter the results. For most identified proteins, a localization probability threshold of ≥ 75% was applied to assign AMPylation sites, consistent with commonly accepted high-confidence standards in proteomic analyses. For ATP synthase subunits and Trx3, a more permissive threshold (≥50%) was accepted, due to our specific interest in ATP synthase regulation and previously published experimental evidence supporting Trx3 modification.^34^ Peptides and respective modification sites are presented in Supplementary Table 3. Part of data was also preprocessed with Mascot Distiller (version 2.8, Matrixscience) and searched with Mascot search engine (version 2.8, Matrixscience) against the SGD database (version 20210423; 6624 protein sequences), with offline mass recalibration (MScan software, version 3.0, https://proteom.ibb.waw.pl/mscan/) based on results of preliminary Mascot searches, typical resulting parent mass window of 5 ppm and fragment mass window of 0.01 Da. The rest of the search parameters were similar to the ones used in Proteome Discoverer searches. Results were validated using a target/decoy strategy, with a threshold q value of 0.01.

### High-Confidence Mitochondrial Proteome and Functional Enrichment

To define a high-confidence mitochondrial proteome, we first screened and compared the proteins identified in each experiment with previously reported mitochondrial proteins from six large-scale studies aimed at characterizing the complete yeast mitochondrial proteome.^54^ This list was again validated against the curated list of yeast mitochondrial proteins in the SGD database. In addition, we analyzed the identified proteins using three different predictive analyses, determining mitochondrial protein localization and the presence of mitochondrial import sequences.^55^ To investigate the functional roles of the identified proteins, pathway and process enrichment analysis were performed. To investigate the enriched pathways of the identified proteins, protein interaction networks were constructed using Metascape.^56^ To map the relationships between enriched terms, a network graph was constructed using a curated subset. This subset was derived from selecting the most significant terms (best p-values) from 20 clusters, applying a limit of 15 terms per cluster and 250 terms overall. The resulting network was visualized in Cytoscape, where each term is a node. Edges connect nodes that share a similarity score greater than 0.3 with nodes colored by their cluster ID.

### Measurement of Oxygen Consumption, ATP Synthesis, and Membrane Potential

Mitochondria were isolated from cells grown in SC-glycerol-ura medium at 28 °C according to the protocol described above, but without their purification by percol gradient centrifugation. For all assays, they were diluted to 75 μg/mL in respiration buffer (10 mM Tris-maleate, pH 6.8, 0.65 M mannitol, 0.35 mM EGTA, and 5 mM Tris-phosphate). Oxygen consumption rates were measured using a Clarke electrode, adding consecutively 4 mM NADH (state 4 respiration), 150 μM ADP (state 3), or 4 μM carbonyl cyanide m-chlorophenylhydrazone (CCCP, uncoupled respiration), as described previously.^57^ To measure complex IV activity, CCCP was added first to mitochondria to uncouple respiration, then, simultaneously, 100 μM ascorbate and 3 μM *N*,*N*,*N*′,*N*′-Tetramethyl-*p*-phenylenediamine (TMPD) were added, and oxygen consumption rates were measured. The rates of ATP synthesis were determined under the same experimental conditions with 750 μM ADP. Every 15 s, 100 μL aliquots were withdrawn from the oxygraph cuvette and added to 50 μL of the 3.5% (w/v) perchloric acid and 12.5 mM EDTA solution previously prepared in the tubes to stop the reaction. The samples were then neutralized to pH 6.5 by the addition of KOH and 0.3 M MOPS. The synthesized ATP was quantified using a luciferin/luciferase assay (Kinase-Glo Max Luminescence Kinase Assay, Promega) in a Beckman Coulter Paradigm plate reader. Variations in transmembrane potential (ΔΨ) were evaluated by monitoring the fluorescence quenching of Rhodamine 123 (0.5 μg/mL; λ_exc_ of 485 nm and λ_em_ of 533 nm) from mitochondrial samples (0.150 mg/mL) in the respiration buffer under constant stirring at 28 °C using a Cary Eclipse Fluorescence Spectrophotometer (Agilent Technologies, Santa Clara, CA, USA) as described previously.^58^ The experiment was performed twice.

### Mitochondrial Membrane Potential Measurement *In Vivo*

Yeast cells were grown to logarithmic phase overnight in SC-glucose-ura 28 °C. Two OD of yeast cells were pelleted down, washed with medium, and incubated in the same growth media containing 100 nM of MitoTracker Red CMXRos (Life Technologies) for 30 min at room temperature. Half of the control cell culture was passed to another tube, and CCCP was added to a final concentration of 25 μM. After 10 min of shaking the MitoTracker Red CMXRos was added without washing off CCCP. After staining, cells were spun down and resuspended in the same media, and only one image was taken immediately by fluorescence microscopy, as the fluorescence signal dropped very fast with time. Multiple technical repetitions were performed until the appropriate number of photographed cells for statistics was obtained.^59^ The experiment was performed with four biological replicates. Images were collected using a Nikon C1 confocal scanning head mounted on an Eclipse TE2000-E inverted microscope equipped with CFI Plan Apochromat VC 100x oil objective (NA 1.4). MitoTracker CMXRos fluorescence was excited with green light emitted by a He–Ne Laser 543 nm laser (Melles Griot, 1.0 mW) set at 3%, then collected with a 610 long pass emission filter and displayed in false 16 colors (LUTs). In all experiments, the acquisition settings, such as the laser power, the gain of the PMT, the pixel dwell, and the pinhole size, were the same. Reference DIC images were collected concomitantly by the detector. A stack of confocal sections (each consisting of 10 optical sections with 0.5 μm intervals) was collected in Nikon EZ-C1 software. The collected z-stacks were processed by the 3D Deconvolution module in NIS-AR 6.02.03 (Nikon) and digitally processed using FIJI software (Fiji Is Just ImageJ 2.14.0; Java 1.8.0–322 [64-bit]). All presented images are the maximum intensity z-projections and were compiled in the FigureJ plugin.^60^ To compare levels of the mitochondrial membrane potential in the tested strains, the fluorescence intensity was quantified using FIJI. Z-max projections obtained from the deconvoluted stacks were segmented using Otsu’s automatic thresholding method. The accuracy of the mask was visually checked to ensure that the selected area corresponded to the mitochondria, and additional manual selection was introduced. Mean fluorescence intensity was measured in the individual region of interest (ROI) and specifies the net of mitochondria per cell.

### Data and Statistical Analyses

Unless otherwise stated in the figure legends or method section, each experiment was repeated at least three times. Data are presented as a representative experiment or as the average ± s.d. To assess significant differences between the control and test samples, the unpaired student’s *t* test was used. The Kruskal–Wallis statistical tests were performed with GraphPad Prism (10.4.0; San Diego, CA, USA) to quantify the statistics of microscopic data. The structure of Atp16 was analyzed from the AlphaFold Protein Structure Database (AF-Q12165-F1-v4) and the structural visualization was carried out using PyMOL software. Images were processed using Adobe Photoshop (Adobe Inc.), and figures were assembled using Adobe Photoshop or Microsoft Office PowerPoint. The mass spectrometry proteomics data have been deposited to the ProteomeXchange Consortium via the PRIDE^61^ partner repository with the data set identifier PXD058585 and 10.6019/PXD058585.

## Results

### Defining a High-Confidence Yeast Mitochondrial Proteome

Knowing that Fmp40 is a mitochondrial protein highly expressed in respiring cells, involved in regulation of redox homeostasis in the cell, and that cells lacking *FMP40* grow slower in liquid medium with a nonfermentable carbon source glycerol at elevated temperature (Figure S1A), we employed several approaches to identify mitochondrial AMPylated proteins (Figure 1A). Starting with wild-type and *fmp40*Δ strains grown in glycerol-containing medium, we first isolated and purified mitochondria using differential centrifugation followed by Percoll gradient centrifugation, a protocol we have previously applied and validated.^62^ Second, as the slower growth on glycerol medium indicates reduced activity of the respiratory chain and/or ATP synthase, the ATP synthase dimers and monomers were either separated by native gel electrophoresis or subjected to affinity chromatography using a strain expressing *ATP6-HA-6xHis* from the mitochondrial genome, and subsequently, the subunits of the purified enzyme were separated by SDS-PAGE (Figure S1B,C). The bands containing ATP synthase dimers, monomers, subunits, and copurified associated proteins were cut from the gels, subjected to in-gel digestion, and analyzed by LC-MS. Third, we purified proteins associated with Fmp40-Myc, and a similar approach was used to purify the mitochondrial redoxin Trx3-Myc along with its associated proteins, since previous studies from our group identified Trx3 as one of Fmp40s substrates.^34^ Using the criteria described in the Methods section, we identified 1322 proteins as constituting a high-confidence mitochondrial proteome. We further classified proteins as authentic mitochondrial proteins if they were absent from literature or previous studies but were identified both in Percoll-purified mitochondria and in at least one additional analysis—either from ATP synthase dimer and monomer isolated via native gels or from Trx3-Myc pull-down assays. Proteins identified only in Percoll-purified mitochondria or only in other pull-down analyses (but not both) were excluded. This approach yielded an additional 398 proteins, bringing the total number of proteins identified as present in yeast mitochondria to 1720 (Table S1). Protein classification was performed using Metascape^56^ (Figure 1B) and visualized by Cytoscape as outlined in the methods section.

**Figure 1:**
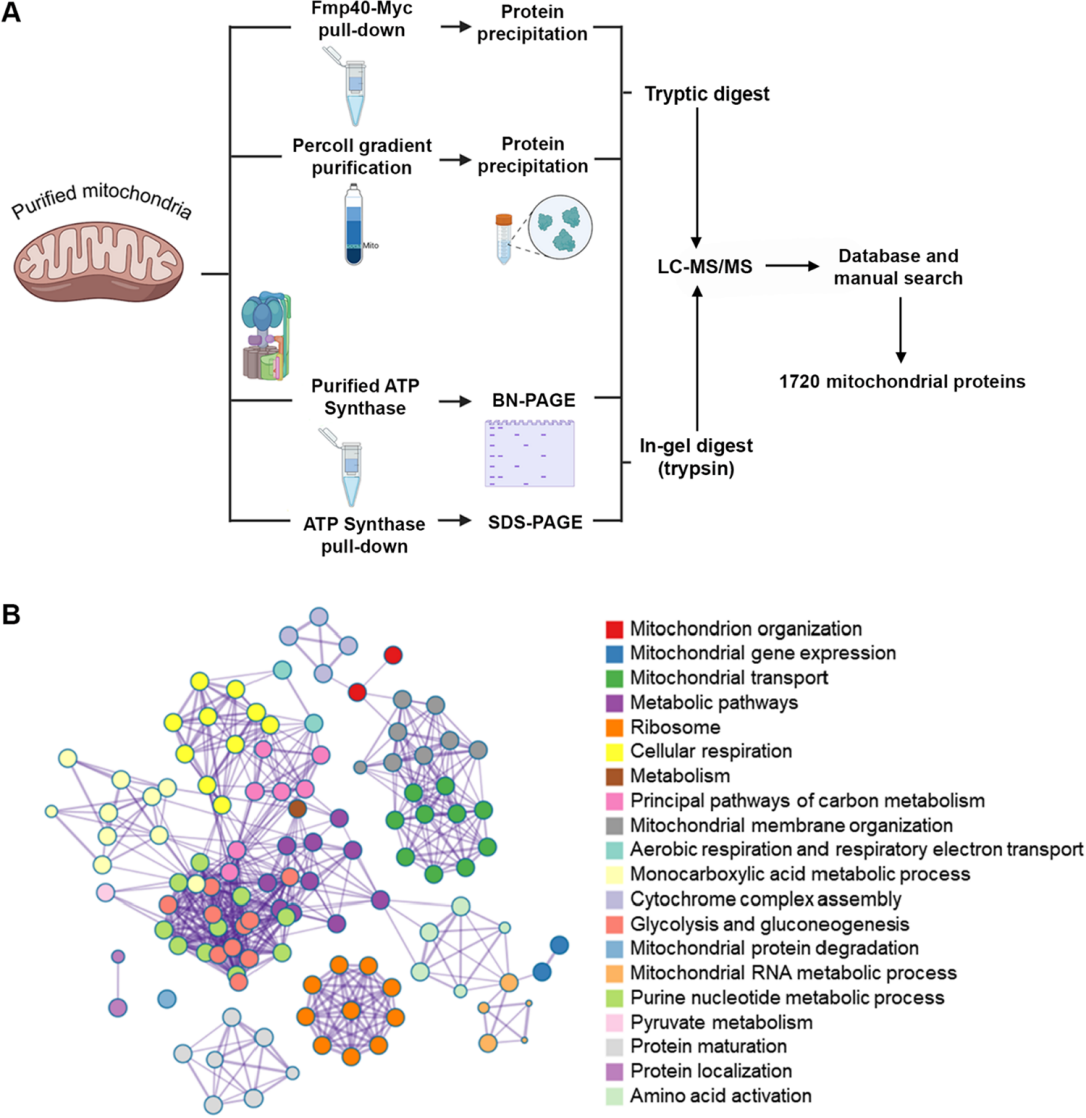
(A) Scheme of experimental approaches for identification of mitochondrial AMPylated proteins. Starting with wild-type and *fmp40*Δ yeast mitochondria, several parallel approaches are outlined in the figure: purification of mitochondria by Percoll gradient, pull-down of Fmp40 or Trx3 and associated proteins, extraction of ATP synthase complexes for BN-PAGE separation, pull-down of ATP synthase, and SDS-PAGE separation of its subunits (from the wild-type cells) followed by subsequent LC-MS/MS analysis. The technical details are described under “Experimental procedures.” (B) Functional classification of identified mitochondrial proteins using Metascape. The top 20 most significantly enriched clusters are depicted in the cluster, with terms exhibiting a high similarity score connected by edges in the network. The nodes are colored by their cluster ID (see methods for details).

### Identification of AMPylation Sites in Yeast Mitochondrial Proteins

AMPylation sites described in this section were identified through a multistage filtering process, as detailed in the Experimental Procedures section, with a final modification-site probability threshold of 75%, consistent with standards commonly applied in high-throughput analyses. However, a more permissive site probability cutoff of 50% was accepted for proteins of particular interest, namely ATP synthase subunits and the previously identified Fmp40 substrate Trx3,^34^ resulting in the identification of three additional putative modification sites for future investigation (Supplementary Table S3). In total, we identified 173 distinct AMPylation sites in 167 peptides derived from 124 proteins, which were classified according to the criteria described above for the mitochondrial proteome. Among these proteins, 108 contained a single AMPylation site, 8 contained two sites, 6 contained three sites, and 1 contained four AMPylation sites. Notably, five or more AMPylation sites were found in four proteins: five in Atp1, Hsp60, and Ssc1, seven in Atp2. We identified the following AMPylated residues with high (probability score ≥ 75) confidence: 46 AMP-serine (S), 32 AMP-threonine (T), 17 AMP-tyrosine (Y), 8 AMP-histidine (H), and 57 AMP-lysine (K) residues. Additionally, 71 peptides were found exclusively in samples from the wild-type strain, but were absent in *fmp40*Δ cells. Among these, 17 modifications were of serine (35% of all serine modifications), 10 of threonine (30% of all threonine modifications), and 4 of tyrosine (24% of all tyrosine modifications). These modifications, potentially dependent on the Fmp40 AMPylase, were found in proteins such as Atp1, Atp2, Atp7, Atp16, Coq5, Eno2, Hsp60, Mia40, Nde1, Ndi1, Sam1, Sam2, Ssa2, Ssb2, Tdh3, Tef2, Trx3, Vps1, and Vps7. However, ∼69% of serine, threonine, and tyrosine residue modifications were also present in samples from *fmp40*Δ cells, suggesting the involvement of other AMPylases in yeast mitochondria specific to these residues, independent of AMPylases targeting histidine or lysine residues. The identified AMPylation sites are summarized in Table 2 and Supplemental Tables S2 and S3.

**Table 2:** AMPylated Peptides in Purified Yeast Mitochondria Identified in this Study

	ORF	Gene	Protein function	Identified AMP-peptide	Position of the AMPylated amino acid
1	*YMR072W*	*ABF2*	Uncharacterized, mtDNA-binding protein	[K].EFDE**K**LPPKKPAGPFIK.[Y]^a^	K-113
2	*YBL015W*	*ACH1*	Uncharacterized protein with CoA transferase activity	[E].DG**S**IVPGPSVGGSPEFITVSDK.[V]^a^	S-155
				[M].**H**APSARPTK.[V]^a^	H-410
				[L].DILV**T**DQGLADLR.[G]^a^	T-446
				[S].IPVDPE**K**VVAIVESTMR.[D]^a^	K-224
3	*YLR304C*	*ACO1*	Aconitase, TCA cycle	[S].KM**K**EVAVANNWPLDVR.[V]^a^	K-362
4	*YAL054C*	*ACS1*	Acetyl-coA synthetase isoform	[S].**Y**IENHSLK.[S]	Y-427
5	*YMR303C*	*ADH2*	Alcohol dehydrogenase II	[G].GA**H**GIINVSVSEAAIEASTR.[Y]	H-241
6	*YJL131C*	*AIM23*	Mitochondrial translation initiation factor 3 (IF3, mIF3)	[A].**H**EIVSLLK.[K]^a^	H-232
7	*YER080W*	*AIM9*	Unknown mitochondrial protein	[T].V**S**LVNNVVPK.[N]^a^	S-120
8	*YOR374W*	*ALD4*	Mitochondrial aldehyde dehydrogenase	[D].EVINMAND**S**EYGLAAGIHTSNINTALK.[V]^a^	S-445
9	*YPL061W*	*ALD6*	Cytosolic aldehyde dehydrogenase	[E].PVKITLPNGL**T**YEQPTGLFINNK.[F]^a^	T-21
10	*YGL148W*	*ARO2*	Bifunctional chorismate synthase and flavin reductase	[D].ASVAGLMV**K**EIEK.[Y]	K-203
11	*YBL099W*	*ATP1*	ATP synthase subunit α	[N].IQAEELVEFSSGV**K**GMALNLEPGQVGIVLFGSDR.[L]^a^	K-97
				[L].SLLLRRPPGREA**Y**PGDVFYLHSR.[L]^a^	Y-331
				[K].LSEKEGSGSLTALPVIE**T**QGGDVSAY.[I]	T-366
				[P].LI**Y**AGVNGHLDGIELSR.[I]/[S].PLA**T**EEQVPLI**Y**AGVNGHLDGIELSR.[I]^a^	Y-483
				[E].L**S**RIGEFESSFLSYLK.[S]	S-496
12	*YJR121W*	*ATP2*	ATP synthase subunit β	[R].CMA**S**AAQSTPITGK.[V]^a^	S-35
				[K].TPQG**K**LVLEVAQHLGENTVR.[T]^a^	K-78
				[D].**T**GGPISVPVGR.[E]^a^	T-112
				[R].E**T**LGRIINVIGEPIDERGPIK.[S]	T-124
				[A].GVG**K**TVFIQELINNIAK.[A]^a^	K-196
				[V].A**S**KVQETLQTYK.[S]	S-405
				[N].IPE**H**AFYMVGGIEDVVAK.[A]	H-488
13	*YBR039w*	*ATP3*	ATP synthase subunit γ	[K].ISIF**Y**NDPVSSLSFEPSEKPIFNAK.[T]^a^	Y-199
14	*YDR298C*	*ATP5*	ATP synthase subunit OSCP	[N].P**K**LGHLLLNPALSLKDR.[N]^a^	K-69
15	*YKL016C*	*ATP7*	ATP synthase subunit *d*	[S].VL**K**NTSVIDKIESYVK.[Q]	K-64
16	*YPL271W*	*ATP15*	ATP synthase subunit ε	[Y].**K**NGTAASEPTPITK.[A]^a^	K-49
17	*YDL004W*	*ATP16*	ATP synthase subunit δ	[A].**S**SGLKLQFALPHETLYSGSEVTQVNLPAK.[S]	S-29
				[R].IGVLANHVPTVEQLLPGVVEVMEG**S**N.[S]	S-85
28	*YML042W*	*CAT2*	Carnitine acetyl-CoA transferase	[N].RFYDK**S**LQFLVTGNGSSGFLAEHSK.[M]^a^	S-361
19	*YER168C*	*CCA1*	ATP (CTP):tRNA-specific tRNA nucleotidyltransferase	[L].NAP**K**ITLTK.[V]	K-29
20	*YAL038W*	*CDC19*	Pyruvate kinase	[R].**K**AGLNIVR.[M]	K-42
21	*YNR001C*	*CIT1*	Mitochondrial citrate synthase	[E].LF**K**LVSTIYEVAPGVLTK.[H]	K-386
22	*YCR005C*	*CIT2*	Peroxisomal citrate synthase	[Y].LSLA**S**GLNGLAGPLHGR.[A]^a^	S-283
				[L].LDNLP**K**DLHPMAQFSIAVTALESESK.[F]^a^	K-153
23	*YML110C*	*COQ5*	2-hexaprenyl-6-methoxy-1,4-benzoquinone methyltransferase; involved in ubiquinone (Coenzyme Q) biosynthesis	[F].IDVAGG**S**GDIAFGLLDHAESK.[F]	S-122
24	*YLR290C*	*COQ11*	Putative oxidoreductase, subunit of Coenzyme Q complex	[R].LL**S**FGAGPNPLKK.[S]^a^	S-107
25	*YGL119W*	*COQ8*	ATPase required for ubiquinone biosynthesis	[I].DSDLN**S**LLMLLTASSLLPK.[G]^a^	S-232
26	*YBL045C*	*COR1*	Core subunit of the ubiquinol-cytochrome c reductase complex	[K].**K**IDAITVKDVK.[A]	K-409
27	*YPR124W*	*CTR1*	High-affinity copper transporter of plasma membrane	[P].TQEEGCNCA**T**DSGK.[N]	T-367
28	*YML054C*	*CYB2*	Cytochrome b2 (L-lactate cytochrome-c oxidoreductase)	[F].V**T**VDAPSLGQR.[E]^a^	T-360
29	*YAL039C*	*CYC3*	Cytochrome c heme lyase	[F].MG**K**PGVLSPR.[A]	K-162
30	*YOR204W*	*DED1*	ATP-dependent DEAD-box RNA helicase	[V].V**Y**GGSPIGNQLR.[E]	Y-260
31	*YHR011W*	*DIA4*	Probable mitochondrial seryl-tRNA synthetase	[L].EEI**K**QFQISVVEELGIPAK.[V]^a^	K-325
32	*YDL174C*	*DLD1*	Major mitochondrial D-lactate dehydrogenas	[G].**K**RE**Y**LLEELGEAPVDLMR.[K]^a^	K-540, Y-543
33	*YDL178W*	*DLD2*	D-2-hydroxyglutarate dehydrogenase	[E].AELVGD**S**PKPVVGAIGYGHVGDGNLHLNVAVR.[E]^a^	S-435
34	*YHR174W*	*ENO2*	Enolase II, a phosphopyruvate hydratase	[F].F**KT**AGIQIVADDLTVTNPAR.[I]	K-312, T-313
35	*YML012W*	*ERV25*	Member of the p24 family involved in ER to Golgi transport	[S].L**S**RAIELDIESGAEAR.[D]	S-112
36	*YPL222W*	*FMP40*	Mitochondrial ampylase	[A].RDLELLL**K**GELNSVDDALEK.[T]	K-409
37	*YDR019C*	*GCV1*	T subunit of the mitochondrial glycine decarboxylase complex	[A].**K**MPLVPTHYYKQ.[-]^a^	K-389
38	*YCL040W*	*GLK1*	Glucokinase	[S].MLR**H**ALALSPLGAEGER.[K]	H-465
39	*YPR160W*	*GPH1*	Glycogen phosphorylase required for mobilization of glycogen	[T].**T**NDMI**T**EEPTSPHQIPR.[L]^a^	T-9, T-14
40	*YKL152C*	*GPM1*	Tetrameric phosphoglycerate mutase	[N].E**K**NLFTGWVDVK.[L]^a^	K-17
				[Y].RR**S**FDVPPPPIDASSPFSQK.[G]^a^	S-116
41	*YIL155C*	*GUT2*	Mitochondrial glycerol-3-phosphate dehydrogenase	[V].VNA**T**GPYSDAILQMDR.[N]	T-284
42	*YDR232W*	*HEM1*	5-aminolevulinate synthase	[V].IPNP**S**HIVPVLIGNADLAK.[Q]^a^	S-417
43	*YLR259C*	*HSP60*	Tetradecameric mitochondrial chaperonin	[K].VEFE**K**PLLLLSEKKISSIQDILPALEISNQSR.[R]^a^	K-238
				[P].**K**GRNVLIEQPFGPPK.[I]^a^	K-55
				[R].SGLVDASGVASLLAT**T**EVAIVDAPEPPAAAGAGGMPGGMPGMPG.[M]^a^	T-542
				[S].LL**K**GVETLAEAVAATLGPK.[G]^a^	K-409
				[E].DELEV**T**EGMRFDRGFISPYFITDPK.[S]	T-211
44	*YDR258C*	*HSP78*	Oligomeric mitochondrial matrix chaperone	[A].**K**ANLEQAR.[I]	K-411
45	*YER065C*	*ICL1*	Isocitrate lyase; catalyzes the formation of succinate and glyoxylate from isocitrate	[I].ER**S**ALSNKQELIK.[K]	S-330
46	*YNL037C*	*IDH1*	Subunit of mitochondrial NAD(+)-dependent isocitrate dehydrogenase	[K].**T**RIPDIDLIVIR.[E]^a^	T-129
47	*YOR136W*	*IDH2*	Subunit of mitochondrial NAD(+)-dependent isocitrate dehydrogenase	[F].GLFANVRPA**K**SIEGFKTTYENVDLVLIR.[E]^a^	K-138
48	*YMR108W*	*ILV2*	Acetolactate synthase	[V].L**Y**VGAGILNHADGPR.[L]	Y-305
49	*YLR355C*	*ILV5*	Acetohydroxyacid reductoisomerase and mtDNA binding protein	[T].**H**VEPPKDLDVILVAPK.[G]^a^	H-182
				[P].VF**K**DLTHVEPPKDLDVILVAPK.[G]^a^	K-178
				[P].G**K**NLFTVEDAIKR.[G]^a^	K-126
50	*YDR332W*	*IRC3*	Double-stranded DNA-dependent helicase of the DExH/D-box family	[T].GGG**K**TVIFSNLINQLR.[Q]^a^	K-65
51	*YIL125W*	*KGD1*	Subunit of the mitochondrial α-ketoglutarate dehydrogenase complex	[K].ELG**K**VLSSWPEGFEVHK.[N]^a^	K-614
				[C].NFQVV**Y**PTTPANLFHILR.[R]^a^	Y-822
52	*YHR155W*	*LAM1*	Putative sterol transfer protein	[S].LTVPDLNLL**T**RK.[M]	T-1123
53	*YNL045W*	*LAP2*	Leucyl aminopeptidase yscIV with epoxide hydrolase activity	[V].F**S**QLEAIHAR.[S]	S-183
54	*YNL071W*	*LAT1*	Dihydrolipoamide acetyltransferase component (E2) of the PDC	[D].V**S**VAVATPTGLLTPIVK.[N]	S-345
				[S].IIGERLLQ**S**TQGIPSYIVSSK.[I]^a^	S-270
55	*YMR296C*	*LCB1*	Component of serine palmitoyltransferase;	[V].**T**MEMPIQNHITITR.[N]^a^	T-124
56	*YGL009C*	*LEU1*	Isopropylmalate isomerase	[K].DFGI**K**SIIAPSYGDIFYNNSFK.[N]	K-644
57	*YNL104C*	*LEU4*	α-isopropylmalate synthase	[F].A**T**INNIIHSGDVSIPSLAEVEGK.[N]^a^	T-590
58	*YOR108W*	*LEU9*	α-isopropylmalate synthase II	[V].IMR**S**LGLDVPRPMQVDFSNTLQK.[N]^a^	S-428
59	*YFL018C*	*LPD1*	Dihydrolipoamide dehydrogenase	[R].ILGAHIIGPNAGEMIAEAGLALE**Y**GA**S**.[A]^a^	Y-464, S-467
				[R].ILGAHIIGPNAGEMIAEAGLALEYGA**S**AED.[V]^a^	S-467
				[K].TNKQENLEAEVLLVAVGRRP**Y**IAG.[L]^a^	Y-309
60	*YGR244C*	*LSC2*	β subunit of succinyl-CoA ligase	[I].HE**Y**RSAQLLREYGIGTPEGFPAFTPEEAFEAAK.[K]^a^	Y-36
61	YIL094C	*LYS12*	NAD-linked mitochondrial homoisocitrate dehydrogenase; required for the fourth step in the biosynthesis of lysine	[P].IATIRSTALMLEFLG**H**NEAAQDIYK.[A]^a^	H-329
62	YKL029C	*MAE1*	Mitochondrial malic enzyme	[T].ITD**K**MISAAVDQLAELSPLR.[E]^a^	K-562
63	*YNL307C*	*MCK1*	Dual-specificity ser/thr and tyrosine protein kinase	[H].PNIV**K**LQYFFTHLSPQDNK.[V]	K-94
64	*YKL150W*	*MCR1*	Mitochondrial NADH-cytochrome b5 reductase	[L].L**Y**GNKTPQDILLR.[K]^a^	Y-195
				[G].INPL**Y**QLAHHIVENPNDK.[T]^a^	Y-175
65	*YKL085W*	*MDH1*	Mitochondrial malate dehydrogenase	[A].**K**FANAVLSGFKGER.[D]^a^	K-251
				[G].A**K**FANAVLSGFK.[G]^a^	K-251
				[F].**K**GERDVIEPSFVDSPLFK.[S]	K-261
66	*YPL270W*	*MDL2*	Mitochondrial inner membrane half-type ABC transporter	[T].**S**LPSASPISK.[G]^a^	S-76
67	*YOL027C*	*MDM38*	Membrane-associated mitochondrial ribosome receptor	[P].**T**STDAPAKPK.[E]^a^	T-71
68	*YKL195W*	*MIA40*	Import and assembly protein of IMS	[E].QSNEDNATANNQ**K**DENISSENSEENTSDKTLDNNAGSSEK.[K]^a^	K-181
				[K].SDENGDDND**S**KNDETEAGPQLGGDKIGASK.[V]	S-114
				[V].N**T**IESAPNVSSAK.[E]^a^	T-369
69	*YJR077C*	*MIR1*	Mitochondrial phosphate carrier	[G].LVGGFSRIL**K**EEGIGSFYSGFTPILFK.[Q]^a^	K-162
70	*YLR190W*	*MMR1*	Unknown protein of the mitochondrial outer membrane	[T].GPIGN**S**LLYPTSLSK.[L]^a^	S-51
71	*YPL118W*	*MRP51*	Mitochondrial ribosomal protein of the small subunit	[H].PT**H**QIIETKPSTLYR.[Q]^a^	H-33
				[Q].IIE**T**KPSTLYR.[Q]^a^	T-38
72	*YNL005C*	*MRP7*	Mitochondrial ribosomal protein of the large subunit	[I].G**K**DHSIFALEPGVVR.[Y]^a^	K-81
73	*YDL202W*	*MRPL11*	Mitochondrial ribosomal protein of the large subunit	[A].**T**ITYEDTNPQQVAK.[L]^a^	T-139
74	*YKL170W*	*MRPL38*	Mitochondrial ribosomal protein of the large subunit	[R].KDGSTVAFGDTACVLINKN**T**GEPL.[G]^a^	T-103
75	*YJL063C*	*MRPL8*	Mitochondrial ribosomal protein of the large subunit	[A].KF**K**AEAQLHGEIMLIK.[Q]^a^	K-177
76	*YNL306W*	*MRPS18*	Mitochondrial ribosomal protein of the small subunit	[S].**S**EVYKPK.[E]	S-64
77	*YGR165W*	*MRPS35*	Mitochondrial ribosomal protein of the small subunit	[I].FEEI**T**VEGLSAQQVSQK.[Y]^a^	T-162
78	*YDR194C*	*MSS116*	Mitochondrial transcription elongation factor	[T].LD**S**LLEEGVLDKEIHK.[A]	S-106
79	*YLR203C*	*MSS51*	Specific translational activator for the mitochondrial COX1 mRNA	[Y].**T**RSFYSMDTEFQLAAVTK.[M]^a^	T-167
80	*YPL097W*	*MSY1*	Mitochondrial tyrosyl-tRNA synthetase	[N].VA**S**ISQQLQR.[F]^a^	S-155
81	*YML031W*	*NDC1*	Subunit of the transmembrane ring of the nuclear pore complex (NPC)	[L].NLV**S**LLIIVTR.[K]^a^	S-74
82	*YMR145C*	*NDE1*	Mitochondrial external NADH dehydrogenase	[T].**T**LYNVVVVSPR.[N]	T-134
83	*YML120C*	*NDI1*	NADH:ubiquinone oxidoreductase	[S].**S**YAQSHLENTSIK.[V]	S-287
84	*YJL208C*	*NUC1*	Major mitochondrial nuclease	[V].AEAP**T**ANPAREDIAVAAFVLPNEPISNETK.[L]^a^	T-239
85	*YPR002W*	*PDH1*	Putative 2-methylcitrate dehydratase	[Q].NII**K**PIVPGTIVPSGTK.[I]^a^	K-87
86	*YGR193C*	*PDX1*	E3-binding protein of the mitochondrial pyruvate dehydrogenase complex	[N].LPE**K**NEYILALNVSVNNK.[K]^a^	K-374
				[A].NLEQTLLP**S**VSLLLAENNISK.[Q]^a^	S-173
87	*YBR196C*	*PGI1*	Glycolytic enzyme phosphoglucose isomerase	[K].PM**Y**VDGVNVAPEVDSVLK.[H]	Y-121
88	*YPL036W; YGL008C*	*PMA2*	Plasma membrane H^+^-ATPase; isoform of Pma1p	[K].KADTGIAVEGATDAAR**S**AADIVFLAPGL**S**AIID.[A]^a^	S-660, S-672
89	*YNL055C*	*POR1*	Mitochondrial porin	[Q].T**K**LEFANLTPGLK.[N]^a^	K-84
				[A].**Y**KQLLRPGVTLGVGSSFDALK.[L]^a^	Y-247
				[Q].PV**K**DGPLSTNVEAK.[L]^a^	K-50
90	*YNL169C*	*PSD1*	Phosphatidylserine decarboxylase of the mitochondrial inner membrane	[A].SPSDG**K**ILQVGIINSETGEIEQVK.[G]^a^	K-212
91	*YAL043C*	*PTA1*	Subunit of holo-CPF, a complex required for the cleavage and polyadenylation of mRNA and snoRNA 3′ ends	[Q].**T**LHVIGEETK.[S]	T-322
92	*YFR033C*	*QCR6*	Subunit 6 of ubiquinol cytochrome-c reductase (Complex III)	[G].EE**K**EEENGDEDEDEDEDEDDDDDDDEDEEEEEEVTDQLEDLREHFK.[N]^a^	K-43
93	*YJL166W*	*QCR8*	Subunit 8 of ubiquinol cytochrome-c reductase (Complex III)	[A].Q**K**PLQGIFHNAVFNSFR.[R]^a^	K-35
94	*YBR191W; YPL079W*	*RPL21A*	Ribosomal 60S subunit protein L21A	[T].K**S**SVGVIINK.[M]^a^	S-70
95	*YPR043W*	*RPL43A*	Ribosomal 60S subunit protein L43A	[G].GA**Y**TVSTAAAATVR.[S]^a^	Y-69
96	*YBR031W; YDR012W*	*RPL4A*	Ribosomal 60S subunit protein L4A	[I].Q**S**AIRPAGQATQK.[R]	S-297
97	*YMR143W*	*RPS16A*	Protein component of the small (40S) ribosomal subunit	[K].GLI**K**VNGSPITLVEPEILR.[F]^a^	K-30
98	*YHL015W*	*RPS20*	Protein component of the small (40S) ribosomal subunit	[F].Q**K**E**K**VEEQEQQQQQIIK.[I]	K-6, K-8
99	*YDR041W*	*RSM10*	Mitochondrial ribosomal protein of the small subunit	[I].**S**LDLGSDANGLEK.[S]^a^	S-149
100	*YLR180W*	*SAM1*	S-adenosylmethionine synthetase	[V].VVV**S**AQHADEITTEDLR.[A]	S-193
101	*YDR502C*	*SAM2*	S-adenosylmethionine synthetase	[T].VVI**S**AQHADEISTADLR.[T]	S-195
102	*YMR214W*	*SCJ1*	One of several homologues of bacterial chaperone DnaJ	[V].L**S**AAEALYGGWQR.[T]	S-299
103	*YJL080C*	*SCP160*	Essential RNA-binding G protein effector of mating response pathway	[E].**T**GEVELEITGSR.[Q]	T-830
104	*YJL045W*	*SDH9*	Minor succinate dehydrogenase isozyme	[G].KN**K**DHILLQLSHLPPEVLK.[E]^a^	K-346
105	*YLR139C*	*SLS1*	Unknown mitochondrial membrane protein	[S].LN**K**IWSNITHENVR.[M]^a^	K-236
106	*YAL005C*	*SSA1*	ATPase involved in protein folding and NLS-directed nuclear transport	[I].**S**EAGDKLEQADKDTVTK.[K]^a^	S-551
107	*YLL024C*	*SSA2*	HSP70 family ATP-binding protein; involved in protein folding	[I].**S**EAGDKLEQADKDAVTK.[K]	S-551
108	*YNL209W; YDL229W*	*SSB2*	Cytoplasmic ATPase, a ribosome-associated molecular chaperone	[A].I**S**GLNVLR.[I]	S-167
109	*YJR045C*	*SSC1*	Hsp70 family ATPase	[I].F**S**TAAAGQTSVEIR.[V]^a^	S-450
				[I].PA**K**RQAVVNPENTLFATK.[R]^a^	K-83
				[E].ARGQ**T**YSPAQIGGFVLNK.[M]^a^	T-137
				[G].IPA**K**RQAVVNPENTLFATK.[R]^a^	K-83
				[T].TIPT**K**KSQIFSTAAAGQTSVEIR.[V]**	K-444
110	*YLR369W*	*SSQ1*	Mitochondrial Hsp70-type molecular chaperone	[L].AVI**T**VPAYFNDSQR.[Q]^a^	T-181
111	*YOR086C*	*TCB1*	Lipid-binding ER protein involved in ER-plasma membrane tethering	[Q].LNIPQLLSGSNLSIGILEITV**K**.[N]	K-393
112	*YKL027W*	*TCD2*	tRNA threonyl-carbamoyl-adenosine dehydratase	[K].KRGIL**S**GIPVVFSAEKPDPK.[K]^a^	S-250
113	*YJL052W*	*TDH1*	Glyceraldehyde-3-phosphate dehydrogenase (GAPDH)	[Q].G**K**LTGMAFRVPTVDVSVVDLTVK.[L]	K-255
114	*YJR009C*	*TDH2*	Glyceraldehyde-3-phosphate dehydrogenase (GAPDH)	[Y].AGEVSHDD**K**HIIVDGHK.[I]^a^	K-63
115	*YGR192C*	*TDH3*	Glyceraldehyde-3-phosphate dehydrogenase (GAPDH)	[L].A**K**VINDAFGIEEGLMTTVHSLTATQK.[T]	K-160
				[E].EGLM**T**TVHSLTATQK.[T]	T-174
116	*YBR118W*	*TEF2*	Translational elongation factor EF-1 α	[V].V**T**FAPAGVTTEVK.[S]	T-277
				[P].LQDV**Y**KIGGIGTVPVGR.[V]	Y-252
				[T].GVI**K**PGMVVTFAPAGVTTEVK.[S]^a^	K-271
117	*YCR083W*	*TRX3*	Mitochondrial thioredoxin;	[K].MMQPHL**T**KLIQAYPDVR.[F]	T-66
118	*YOR187W*	*TUF1*	Mitochondrial translation elongation factor Tu (EF-Tu)	[N].IG**T**IGHVDHGK.[T]^a^	T-53
119	*YKR001C*	*VPS1*	Dynamin-like GTPase required for vacuolar sorting	[L].LKYI**S**KPNAIIL**S**VNAANTDLANSDGLK.[L]	S-207, S-215
120	*YGR234W*	*YHB1*	Nitric oxide oxidoreductase	[S].GIELE**S**LPITPGQYITVNTHPIR.[Q]^a^	S-181
121	*YMR241W*	*YHM2*	Citrate and oxoglutarate carrier protein	[S].ALGGGL**S**AWNQPIEVIR.[V]^a^	S-229
122	*YKR018C*	*YKR018C*	Protein of unknown function, cytoplasm and nucleus	[E].ELIF**T**SVNLLGK.[K]	T-525
123	*YML025C*	*YML6*	Mitochondrial ribosomal protein of the large subunit	[L].I**S**PTPELDLNR.[L]^a^	S-177
124	*YDR349C*	*VPS7*	Putative GPI-anchored aspartic protease	[R].EL**T**IEIPLR.[D]	T-373

a Present in samples from *fmp40*Δ strain mitochondria. AMPylated amino acid residue is indicated by bold font.

### Functional Classification of Identified AMPylated Proteins

We grouped the identified AMPylated proteins based on their determined or proposed functions, as outlined in Table 3 and Table S3, using gene ontology and manual curation. Among other identified AMPylated proteins, 30 are known to bind ATP. Among this protein data set, the two largest groups were formed by proteins involved in mitochondrial energy and carbon metabolism, as well as those involved in various metabolic and biosynthetic pathways. A prominent group within this category consisted of heat shock proteins and those involved in the protein folding pathway, including Hsp60, Hsp78, Ssa1, Ssa2, Ssb2, Ssc1, and Ssq1, having roles in protein processing acting as proteases (Lap2, Vps7), which could be expected given the slower growth of *fmp40*Δ cells at elevated temperature. Another major group of AMPylated proteins belonged to the oxidoreductase family, which included ten members of the flavohemoprotein family, NAD­(P)-binding proteins, and other dehydrogenases, including thioredoxin Trx3 and the nitric oxide oxidoreductase Yhb1, which plays a major role in nitrosative stress response. This suggests that redox proteins are particularly susceptible to AMPylation in mitochondria. Remarkably, 14 subunits of the OXPHOS machinery were also identified as AMPylated, including two subunits of Complex III (Qcr6 and Qcr8), and seven subunits of ATP synthase (Atp1/α, Atp2/β, Atp3/γ, Atp5/OSCP, Atp7/d, Atp15/ε, and Atp16/δ). Also, 14 proteins involved in mitochondrial translation, mitochondrial DNA maintenance (Abf2, Nuc1), ribosome assembly (Mdm38), mitochondrial transport (Mdl2, Mia40, Mir1, Mmr1, Por1, Yhm2), and structural organization of mitochondria (Aim9) were found to be AMPylated. Therefore, AMPylation is widespread among yeast mitochondrial proteins.

**Table 3:** Functional enrichment of AMPylated proteins in mitochondria^a^

**Biosynthesis**	
Amino acid Synthesis	Aro2, Gcv1, Ilv2, Ilv5, Leu1, Leu4, Leu9, Lys12, Sam11, Sam2
Cofactor Synthesis	Coq5, Coq11, Coq8, Hem1
Lipid and Sterol Synthesis	Fas1, Lcb1, Psd1

**Cellular Transport**	
Membrane Transport	Ctr1, *Pma2,* Tcb1
Mitochondrial Transport	Mdl2, Mia40, Mir1, Por1, Yhm2
Nuclear Transport	Ndc1
Vesicular Transport	Erv25, Vps1

**Energy Metabolism**	
Glycolysis	Cdc19, Eno2, Glk1, Gpm1, Pgi1, Tdh1, Tdh2, Tdh3
Metabolism	Ach1, Acs1, Adh2, Ald4, Ald6, Cat2, Cit2, Dld1, Dld2, Gph1, Gut2, Icl1, Mae1, Pdh1
Oxidative Phosphorylation	Atp1, Atp2, Atp3, Atp5, Atp7, Atp15, Atp16, Cor1, Cyb2, Mcr1, Nde1, Ndi1, Qcr6, Qcr8
TCA Cycle	Aco1, Cit1, Idh1, Idh2, Kgd1, Lat1, Lpd1, Lsc2, Mdh1, Pdx1, Sdh9

**Gene Expression** **& Protein Synthesis**	
Cytosolic Translation	Rpl21A, Rpl43A, Rpl4A, Rps16A, Rps20, Tef2
Transcription	Irc3, Mss116
Mitochondrial Translation	Aim23, Dia4, Mrp51, Mrp7, Mrpl11, Mrpl38, Mrpl8, Mrps18, Mrps35, Mss51, Msy1, Rsm10, Tuf1, Yml6
RNA Processing	Cca1, Ded1, Pta1, Tcd2

**Mitochondrial Biogenesis** **& Maintenance**	
Mitochondrial DNA Maintenance	Abf2, Nuc1
Mitochondrial Dynamics and Ribosome Assembly	Mdm38

**Protein Folding** **& Stress Response**	
Chaperone	Hsp60, Hsp78, Mjd1, Scj1, Ssa1, Ssa2, Ssb2, Ssc1, Ssq1
Redox Regulation	Trx3, Yhb1

**Protein Processing** **& Regulation**	
Protease	Lap2, Vps7
Protein Modification and Signaling	Fmp40, Mck1, Psk1, Scp160

**Unknown**	
Unknown mitochondrial protein	Aim9, Mmr1, Sls1
Other	Lam1, Pir1, Ykr018C

a A total of 124 identified mitochondrial AMPylated proteins from the yeast mitochondrial proteome were classified manually and with gene ontology into functional categories.

### New Phosphorylation Sites of Mitochondrial Proteins

Since phosphorylation occurs on the same amino acid residues as AMPylation, the simultaneous identification of phosphopeptides in the same data set provides an additional layer of regulatory context for AMPylated proteins. The raw data from all experiments were also analyzed to identify phospho-peptides, resulting in the detection of 1,540 phosphorylation sites across 516 proteins (Table S4). The list of phosphorylation sites identified in our study was compared to previously reported phospho-sites, as found in the yeast protein phosphorylation database, YeastMine.^63^ The list of 1,077 phosphorylation sites not detected in previous experimental approaches is provided in Table S5 and represents 2.4% of the phosphorylation sites already reported in the yeast proteome. Of the 1720 proteins classified as mitochondrial in this study, 1216 phosphorylation sites were identified in 332 proteins, with 823 of these sites being reported for the first time. Focusing on OXPHOS enzymes, redox enzymes, and subunits of the mitochondrial protein import systems, several previously unreported phospho-sites were identified. Notable examples include Qcr10, Cox5a, Cox9, Cox13, Cox2, Trx3, and Tim23. These findings suggest that the yeast phospho-proteome is still not fully characterized.

### AMPylated and Phosphorylated ATP Synthase Subunits

In further analysis, we focused on ATP synthase, a key enzyme that, in addition to synthesizing cellular energy (ATP), is directly involved in the formation of the mitochondrial mega-channel and the initiation of cell death.^64^ Moreover, dysfunctions of ATP synthase, caused by mutations in the mitochondrial and nuclear genes encoding its subunits, lead to severe, untreatable neurodegenerative diseases.^65^ The production of most ATP synthase subunits is induced during respiration, under the experimental conditions we applied (growth on a nonfermentative carbon source in medium^66^). The 19 AMPylation sites were found across seven ATP synthase subunits (nine of which were detected in data from different experiments, as shown in Table S6, with the majority found in Atp2, Atp1, and Atp16 (Figure S2). Notably, several AMPylation sites were absent in *fmp40*Δ mitochondria, including T-366 and S-496 in Atp1, T-124 and S-405 in Atp2, and S-29, T-42, and S-85 in Atp16. These sites are particularly interesting for further investigation into AMPylation by Fmp40. Because PTMs of the same amino acid residues may have important regulatory significance, we analyzed the phosphorylation of ATP synthase subunits. While 102 phosphorylation sites were previously reported in the literature, we identified 129 phosphorylation sites in our data (Table S6). Of these, 83 phosphorylation sites in ATP synthase subunits were not previously reported (underlined in the last column of Table S6), including 16 in Atp1, 27 in Atp2, 9 in Atp3, 9 in Atp4, 7 in Atp5, 5 in Atp7, 6 in Atp15, and one each in Atp14, Atp18, Atp19, and Atp20. Conversely, 55 phosphorylation sites in ATP synthase subunits were not detected in our data (underlined in the first column of Table S6). Thus, in total, there are 140 phosphorylation sites in ATP synthase subunits identified to date. Of particular interest is that several positions in three ATP synthase subunits are both AMPylated and phosphorylated: T-475 and Y-483 in Atp1, S-35, T-112, T-124, T-351, S-373, and S-405 in Atp2, and S-29 in Atp16. This dual modification suggests a highly sophisticated regulatory mechanism, especially for Atp2, which appears to be the most extensively modified ATP synthase subunit.

### AMPylation of ATP Synthase *In Vivo*

Next, we aimed to check the *in vivo* AMPylation of ATP synthase subunits by Western blotting. After affinity purification of the ATP synthase complex from native mitochondria through Atp6-HA-6xHis subunit and subsequent separation of its subunits by SDS-PAGE, the proteins were transferred onto a nitrocellulose membrane. The membrane was then incubated with an anti-Thr-AMP antibody (clone 7C11,^50^) and with antibodies against ATP synthase subunits, without stripping, in the following order: Atp4, Atp5, Atp16, Atp2, and Atp3 (Figure 2). Five weak, but distinct bands were detected by the anti-Thr-AMP antibody. One band of unknown identity, migrated in the ∼70 kDa region, while two bands in the ∼55 kDa region most likely correspond to the Atp1 and Atp2 ATP synthase subunits (see Figure S1C for separation of ATP synthase subunits under these experimental conditions, stained with silver). The strongest band was observed at ∼30 kDa, but this does not correspond to an ATP synthase subunit. The Atp2 signal (from the anti-Atp2 antibody) overlapped with a lower band of about 55 kDa detected by anti-Thr-AMP. Additionally, a very weak band in the 30–35 kDa region seemed to overlap with a band detected by the anti-Atp3 antibody. The commercially available anti-Tyr-AMP antibody detected one more band in the 35 kDa region in a similar experiment (Figure S3A).

**Figure 2:**
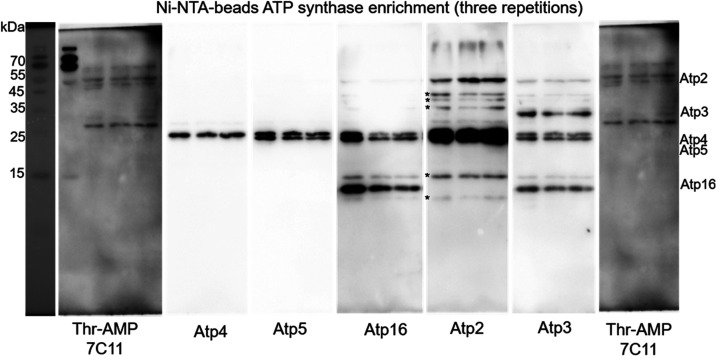
AMPylation of ATP synthase subunits in the affinity-purified enzyme through Atp6-HA-6xHis. Whole ATP synthase complexes were purified from wild-type strain mitochondria using Ni-NTA agarose beads. Two micrograms of Ni-NTA bead eluates were loaded onto a 15% SDS-PAGE gel. After transfer, the membrane was sequentially incubated (without stripping) with the following antibodies: Thr-AMP (clone 7C11,^50^), Atp4, Atp5, Atp2, Atp16, and Atp3. Each lane contains an independent biological replicate; five repetitions were performed, and three are shown. The corresponding silver-stained gel is presented in Figure S1C. Original blots are presented in Figure S5, page S11.

In parallel, ATP synthase complexes were liberated from the inner mitochondrial membrane using the nonionic detergent digitonin and separated on a native gel. The lane fragments containing the monomers and dimers were then transferred onto a second-dimensional denaturing 16% gel. After migration, the proteins were transferred to a membrane. The membrane was first incubated with an anti-Tyr-AMP antibody, then stripped and incubated with an anti-Thr-AMP antibody (commercially available from Merck). The membrane was stripped again and incubated with antibodies against ATP synthase subunits in the following order: Atp4, Atp5, Atp2, Atp16, and Atp3 (Figure S3B). With this approach, the band patterns decorated by both the anti-Tyr-AMP and anti-Thr-AMP antibodies were identical: two bands migrating at 55 kDa, likely corresponding to Atp1 and Atp2, one band at ∼35 kDa, overlapping with Atp3, and three very weak bands in the ∼15 kDa region. The AMP signal in the lanes containing dimers may correspond to Atp20 or Atp21.

### Modification of the Serine Residue at Position 29 in the Atp16 Subunit of ATP Synthase Affects ATP Synthase and OXPHOS Coupling

Within the mitochondrial ATP synthase complex, Atp16 (subunit δ) plays a crucial role in coupling proton translocation with ATP synthesis. In yeast, deletion of the *ATP16* gene results in the inability of yeast to grow on respiratory media and poor growth on fermentative media due to a massive loss of the mitochondrial genome, leading to 100% *petites* cells (ρ^–^/ρ^0^).^67,68^ This defect results from passive proton transport through the enzyme’s proton channel, which is not coupled to ATP synthesis, leading to the loss of the mitochondrial inner membrane potential. The loss of mtDNA serves as a compensatory mechanism, preventing the synthesis of the two mtDNA-encoded subunits, Atp6 and Atp9, which form the proton channel. We therefore hypothesized that the regulation of enzyme coupling at the level of Atp16 might be mediated by the phosphorylation or AMPylation of the serine residue at position 29, as this residue was found to be both phosphorylated and AMPylated in our samples (Figure S2). Serine 29 is located at the beginning of the mature subunit δ, which forms after the cleavage of a 22-amino-acid signal peptide that directs it to the mitochondrial matrix (Figure 3A).^67^ To verify this hypothesis, we introduced codons for alanine or glutamic acid in place of the serine codon at position 29 in the plasmid-borne *ATP16* gene. The plasmids were introduced into wild-type cells, and the *ATP16* gene sequence in the genome was subsequently replaced with the *KANMX4* gene. We assumed that the *ATP16–S29>A/E* variants would be functional and would not cause a massive loss of mtDNA, which was indeed the case. As shown in Figure 3B, the *atp16–S29>A/E* mutations did not impair yeast cell growth on a nonfermentable carbon source. However, when a subinhibitory concentration of ATP synthase inhibitor oligomycin (0.5 μg/mL) was added, it induced slower growth in the yeast *atp16–S29>A/E* cells. Increasing the oligomycin concentration to 0.6 μg/mL completely blocked the respiratory growth of the *atp16–S29>E* mutant. In fermentative growth conditions, mtDNA stability in *atp16*Δ cells was severely reduced (100% of *petite*s cells). In the *atp16–S29>E* cells, the number of *petite* cells reached 30%, compared to 7% in control cells and 12% in *atp16–S29>A* cells. This suggests that the uncoupling of proton translocation and ATP synthesis, along with a decrease in the inner membrane potential (ΔΨ), contributed to the observed phenotypes (Figure 3C).

**Figure 3:**
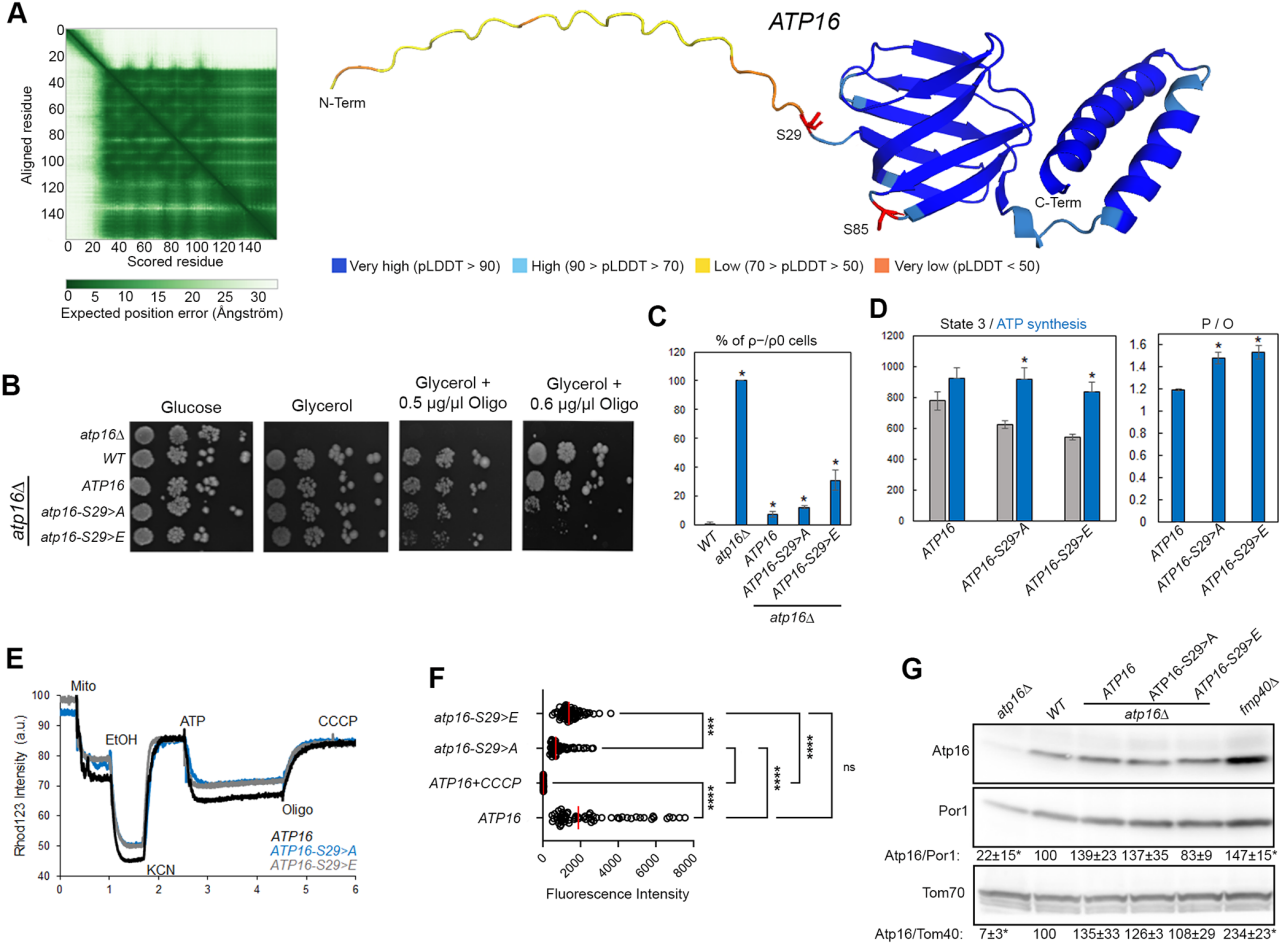
AMPylation/phosphorylation of serine residue at position 29 of subunit δ plays a role in ATP synthase and OXPHOS coupling in both fermentative and respiratory growth conditions. (A) The position of the S29 site in the AlphaFold predicted structure of Atp16 is marked in red. Other AMPylation sites in Atp16 identified in this study are also indicated in red. (B) Respiratory growth: Cells from the indicated strains grown in SC-glucose-ura precultures were serially diluted and spotted onto rich glucose or glycerol plates with or without oligomycin (0.5 or 0.6 μg/mL), and incubated at 28 °C. Plates without oligomycin were scanned after 3 days of incubation, while those with the drug were scanned after 6 days. The experiment was performed twice. (C) Stability of mtDNA: Strains grown overnight in SC-glucose-ura (*atp16*Δ strain with ura) were plated for single colonies on YPGA plates and replicated onto rich glycerol plates to confirm the ρ^+^ vs ρ^®^/ρ^0^ genotype. The percentage of ρ^–^/ρ^0^ colonies was quantified. The graph presents data from the analysis of four biological and three technical replicates for each strain. (D) Oxygen consumption at state 3 and ATP synthesis activities: Oxygen consumption was quantified in nmol O_2_·min^–1^·mg^–1^, and ATP synthesis was measured in nmol ATP·min^–1^·mg^–1^. Oxygen consumption and ATP synthesis values are included in Table S7. (E) Variations in mitochondrial inner membrane potential in isolated mitochondria: The following additions were made: 0.5 μg/mL Rhodamine 123, 150 μg/mL mitochondrial proteins (Mito), 10 μL ethanol (EtOH), 0.2 mM ATP, 2 mM potassium cyanide (KCN), 4 μg/mL oligomycin (oligo), and 4 μM carbonyl cyanide-m-chlorophenyl hydrazone (CCCP). The experiment was performed in two repetitions for each strain. (F) Fluorescence intensity in cells grown in glucose minimal selective medium and stained with MitoTracker CMXRos: Cells were stained with MitoTracker CMXRos (see the Experimental Procedures section for details). Statistical significance of the difference between the control *ATP16*-expressing strain, *ATP16-S29>A/E* gene variants-expressing strains, and CCCP-treated negative control was determined using the Kruskal–Wallis test in GraphPad Prism. Results are presented as a scatter plot of the mean fluorescence intensity value for each count. The experiment was performed in four biological and six technical repetitions for each culture, with 60 cells being quantified. ***p = 0.009 and ****p < 0.0001. (G) The level of Atp16 protein variants in cells grown in nonfermentative medium: Proteins were precipitated using 10% TCA and analyzed by SDS-PAGE (16% SDS-PAGE gel for Atp16 and Por1, and 12% gel run in parallel for Tom70 and Por1) and Western blotting. Protein levels were determined by densitometry and normalized to the respective Por1 signal. The Atp16-S29>E substitution slightly alters protein migration. The statistical significance of changes in plasmid-expressed Atp16 variants or Atp16 in *fmp40*Δ cells compared to control cells was quantified using an unpaired Student’s *t* test based on four biological repetitions. The representative blots are shown. Original blots are presented in the Figure S7, page S13.

To further verify this experimentally, we isolated mitochondria from cells grown in SC-glycerol-ura medium (as the growth in nonfermentative medium was not disturbed and all cells maintained mtDNA) and measured oxygen consumption and ATP synthesis rates. Oxygen consumption with NADH as an electron donor alone (state 4 respiration) was reduced slightly and without statistical significance by 10 ± 9% in *atp16–S29>A* cells and 19.4 ± 12% in *atp16–S29>E* cells compared to the control (Table S7). After successive addition of ADP (state 3, phosphorylating conditions) and CCCP (uncoupled, maximal respiration), statistically significant decreases in oxygen consumption were observed (at state 3:20 ± 7% and 30 ± 6%, uncoupled: 32 ± 4% and 34 ± 7%, respectively, Figure 3D, Table S7). However, the rate of ATP synthesis by ATP synthase remained comparable to that in control cells, leading to an increased ATP production per oxygen atom consumed by the respiratory chain (P/O ratios). Thus, despite the full ATP synthesis activity of ATP synthase, respiratory chain function was reduced in the mutants, in other words, respiratory chain activity is uncoupled from the ATP synthase activity. We conclude from these data that the S29 substitutions lead to subtle passive proton passage through the proton channel within membrane domain of ATP synthase (indicated from the instability of mtDNA in the mutants), and disrupt signaling between ATP synthase and the respiratory chain (as the respiratory chain does not respond with the increase in oxygen consumption at state 4 as observed in *atp16*Δ cells^85^).

To further investigate ATP synthase functionality in the mutant strains, we measured the inner membrane potential changes (ΔΨ) using Rhodamine 123, a cationic dye whose fluorescence is quenched when ΔΨ increases. To evaluate ΔΨ changes due to respiratory chain and ATP synthase activity, the mitochondrial membrane was first energized by feeding electrons from ethanol to the respiratory chain. The resulting ΔΨ was collapsed with the complex IV inhibitor KCN, and ATP was then rapidly added. Under these conditions, the natural ATPase activity inhibitor protein, IF1, is released from the enzyme and does not rebind,^69^ and therefore the inner membrane potential is sustained by the hydrolytic activity of ATP synthase. Ethanol and ATP addition induced a large and stable mitochondrial membrane potential in all strains, which was fully reversed by oligomycin, confirming its dependence on the ATPase activity of ATP synthase (Figure 3E). Despite all cells being ρ^+^ under these experimental conditions, we observed a subtle but consistent decrease in ΔΨ in both the *atp16–S29>A* and *atp16–S29>E* mutants (by approximately 5–10%, depending on the experiment), following both ethanol and ATP addition, which is consistent with lower oxygen consumption at state 4 (Table S7). However, the membrane potential remained stable. Given the significant mtDNA instability observed in the *atp16–S29>A/E* mutants under fermentative growth conditions, we hypothesized that these mutations cause a greater disturbance in ΔΨ when cells are grown on a fermentable carbon source. Since mitochondria are inefficiently isolated from yeast cells grown in glucose-containing media due to glucose repression of mitochondrial biogenesis, we measured ΔΨ in whole cells *in vivo* by fluorescence microscopy. For this, we used the membrane potential-sensitive fluorescent dye MitoTracker Red. When applied at an appropriate concentration, the dye accumulates in mitochondria in a ΔΨ-dependent manner, such that staining intensity positively correlates with the membrane potential.^59^ Wild-type cells treated with CCCP served as a negative control to define mitochondrial-specific MitoTracker Red fluorescence. As expected, the *atp16–S29>A* and *atp16–S29>E* mutants exhibited reduced ΔΨ—approximately 25% and 60% lower than wild-type, respectively (Figure 3F, Figure S4). We also assessed the steady-state levels of Atp16 protein variants (Figure 3G). In cells expressing Atp16 from a plasmid, Atp16 and the Atp16-S29>A variant showed slightly higher protein levels than the genome-encoded Atp16 (“WT” lane), though the difference was not statistically significant. Notably, the Atp16-S29>E variant was not upregulated. Interestingly, deletion of *FMP40* led to a ∼40% increase in endogenously expressed Atp16 levels. In conclusion: (i) Fmp40, along with AMPylation/phosphorylation of serine 29, appears to regulate Atp16 protein levels; (ii) Modification of this residue is directly involved in controlling ATP synthase coupling, as evidenced by ΔΨ reduction and increased frequency of ρ^–^/ρ^0^ cells under fermentative growth, and OXPHOS coupling under respiratory growth (manifested by decreased oxygen consumption relative to ATP synthesis—i.e., increased P/O ratio).

## Discussion

Although AMPylation is not a newly discovered PTM in either prokaryotes or eukaryotes, our understanding of its functional significance remains limited, as both the number of identified AMPylases and confirmed substrate proteins is still relatively small.^21,34,40^ In humans, the only AMPylases identified to date are FicD/HYPE and SelO, and their protein substrates are under active investigation. The list of FicD substrates identified across various experimental approaches and cell lines includes approximately 200 proteins. However, the substrates vary significantly depending on the cell type and experimental conditions.^22,32,35,39,41^ An approach utilizing FicD with a covalently attached and activated nucleotide cosubstrate within its binding site narrowed the list of potential substrates to just eight proteins.^37^ An *in vitro* screen for human SelO substrates identified as many as 124 proteins.^41,42^

Our previous work in yeast identified the mitochondrial redoxins Prx1, Grx2, and Trx3 as *in vitro* substrates of the AMPylase Fmp40, with *in vivo* interactions confirmed for Trx3 and Prx1.^32,34^ In the present study, we systematically profiled AMPylation sites in mitochondrial proteins using a mass spectrometry-based approach. The analysis was conducted on Percoll-purified native yeast mitochondria, isolated ATP synthase complexes, and proteins pulled down with Fmp40-myc and Trx3-myc. We identified 173 distinct AMPylation sites across 124 proteins, of which 95 modifications (55%) occurred on serine, threonine, or tyrosine residues.^70^ The AMPylated proteins identified in our study encompass a wide array of mitochondrial functions, ranging from core bioenergetic processes—including the TCA cycle, oxidative phosphorylation, and pyruvate and carbon metabolism—to redox regulation, mtDNA maintenance and translation, protein folding, and amino acid and lipid biosynthesis. Many heat stress response proteins are AMPylated, which correlates with the slower growth of *fmp40*Δ cells at elevated temperature. This diversity suggests that reversible AMPylation plays a regulatory role across many mitochondrial pathways. Notably, AMPylation was detected on seven ATP synthase subunits, one subunit of the ubiquinol-cytochrome c reductase complex, and 11 TCA cycle enzymes. In contrast, no AMPylated proteins were found among those involved in mitochondrial–nuclear communication. For comparison, previously identified FicD substrates were enriched in proteins related to cellular metabolism and translation, as well as those involved in cytoskeleton structure and signaling, gene expression, ER stress response, and redox homeostasis.^35,39^ Interestingly, many mitochondrial enzymes were also identified as FicD substrates in mammalian cells, despite FicD being localized to the endoplasmic reticulum.^71^ Among the identified proteins are ATP synthase subunits α, β, γ, ε, and homologues of the pyruvate dehydrogenase protein X component (Pdx1 - PDHX), and homologues of NADH dehydrogenase subunits Nde1 and Ndi1, including NDUFA7, NDUFA12, NDUFB10, and NDUFS2. The proteins pulled down with wild-type SelO from human mitochondria include homologues of 24 proteins identified in our yeast experiments.^41^ Among them are the α, β, γ, and OSCP subunits of ATP synthase, mitochondrial citrate synthase (CS), aconitase (ACO2), lactate dehydrogenase (LDHA), glycogen phosphorylase (PYGB), NADH-cytochrome b5 reductase (CYB5R3), NADH oxidoreductase subunit 10 (NDUFA10), the E1 component subunit α of the pyruvate dehydrogenase complex (PDHA1), 2-oxoglutarate/malate carrier protein (SLC25A11), and mitochondrial redoxin PRDX5 (among the seven enzymes that maintain redox homeostasis). These findings suggest that oxidative phosphorylation, along with redox enzymes, is regulated by AMPylation in mammalian cells, similar to what is observed in yeast. The complex II activity is regulated by AMPylation of succinate dehydrogenase subunit A and aconitase by SelO.^41^ Future research will help elucidate how mitochondrial bioenergetics is regulated by AMPylation.

Our analysis identified as many as 96 AMPylated peptides in cells lacking Fmp40. Although no homologues from the FIC family are known in yeast,^25^ the large number of Fmp40-independent modifications suggests the existence of other AMPylases that specifically target serine, threonine, and tyrosine residues, independently of the lysine- and histidine-specific AMPylases. AMPylation modifications of lysine and histidine residues have been reported as transitional modifications.^72,73^ The AMPylation of lysine residues in the catalytic sites of *E. coli* and *Thermococcus barophilus* DNA ligases is considered a transitional step during catalysis.^73,74^ Another enzymes that utilize lysine AMPylation for catalysis are RNA ligases. In humans, the C12orf29 RNA ligase operates via a tandem auto- and RNA-AMPylation mechanism to maintain RNA integrity under ROS-induced cellular stress conditions.^75^ In yeast, the RNA ligase of this family is the Trl1 protein, which was not identified among the AMPylated proteins in our screen.^76^ However, as many as 57 modifications of lysine residues were detected in our data, representing a significant proportion of all AMPylated sites (33%), suggesting that lysine AMPylation is a stable modification. The recent structural discovery of the AMPylase LnaB from *Legionella pneumophila*, where AMP is covalently attached to the catalytic histidine, highlights that AMPylation by LnaB requires auto-AMPylation at the catalytic histidine residue.^77^ LnaB belongs to a distinct family of AMPylases characterized by conserved active site Ser-His-Glu residues. AMPylation of histidine (8 histidine residues identified in our data) and lysine residues seems to be conserved in yeast. However, AMPylases from the LnaB family or enzymes that specifically AMPylate lysine residues have yet to be discovered. Nonenzymatic reactions of ATP or other adenosine-containing nucleotides with proteins could also give rise to these modifications.

Our proteomic methodology also yielded significant insights into the yeast phospho-proteome. Previous studies of the phospho-proteome in yeast cells have demonstrated that subfractionating yeast extracts to purify mitochondria significantly enhances the sensitivity of phospho-protein detection.^12,15,16,18,78,79^ Statistical analysis of available data suggests that the detection of yeast phospho-proteins is nearing saturation.^13,79^ However, we identified 1,077 new phospho-sites across 516 proteins, including 823 novel phospho-sites in 332 mitochondrial proteins. This represents 2.4% of the previously reported phosphorylation sites.^13,15^ Given that more than 50% of these phospho-sites were identified in only one mitochondrial phospho-proteomic study, and that phosphorylation site variation in mitochondrial proteins is influenced by carbon sources,^16^ we propose that the yeast phospho-proteome remains expandable. The phospho-proteomics layer additionally served as an internal indicator of data set depth and PTM coverage. The significant number of previously uncharacterized phosphorylation sites highlights the depth of analysis achieved despite the lack of a PTM enrichment strategy.

Mitochondrial ATP synthase undergoes numerous PTMs. To date, 102 phospho-sites have been identified across 12 different enzyme subunits.^12,13,16,17,80^ In our two approaches to purifying this enzyme, we identified an additional 83 phosphorylation sites in ATP synthase subunits that had not been previously reported, representing 45% of all phospho-sites in the enzyme. Simultaneously, 55 previously identified ATP synthase phosphorylation sites were not detected in our study, which highlights the variability between experiments. This underscores the need for multiple approaches and repetitions to obtain a comprehensive phospho-proteome. The reasons for these discrepancies may include differences in growth conditions, the low stoichiometry of phosphorylation, as well as variations in preparative and instrumental settings in LC-MS experiments. Furthermore, while enrichment of phospho-peptides would have been beneficial for identification of particularly low-abundant entities, it was not applied in this study. Additionally, differences in software for processing mass spectrometry data may also contribute to variability in PTM identification, depending of particular strengths and weaknesses of different algorithms.^81^ The biological significance of ATP synthase subunit Atp2 phosphorylation at T-124 and T-317 has been previously demonstrated.^17^ An increase in the phosphorylation level of Atp2 results in higher abundance and activity of the ATP synthase complex.^17^ Mimicking phosphorylation at position 62 (S62>E) of Atp20 inhibited ATP synthase dimerization, while blocking phosphorylation by substituting alanine for serine actually enhanced the level of dimerization.^12^ Our study extends the scope of ATP synthase regulations to include AMPylation. We report the identification of the AMP moiety at 19 residues across seven ATP synthase subunits. The AMPylation sites were localized at the periphery of the ATP synthase subunits, which aligns with their accessibility to the AMPylating enzyme(s) (Figure 4). Seven AMPylated positions were found to be phosphorylated as well: T-475 and Y-483 in Atp1, S-35, T-112, T-124, and S-405 in Atp2, and S-29 in Atp16. This overlap suggests that AMPylation has the potential to either reversibly or irreversibly mask these phosphorylation sites, further supporting the previously hypothesized crosstalk between these two types of PTM.^82^ Among those doubly modified residues, a few were absent in *fmp40*Δ, indicating that they may be Fmp40-dependent. These residues include: Atp1—T-366 and S-496; Atp2—T-124 and S-405; Atp16—S-29, and S-85. Western blot analysis using the anti-Thr-AMP clone 7C11 ^50^ on ATP synthase complexes pulled down from native mitochondria by affinity purification indicates a high probability of AMPylation of the Atp1 and Atp2 subunits. Since many proteins interacting with ATP synthase are purified during the affinity purification experiment, we employed two-dimensional electrophoresis to filter them. Western blotting with anti-Tyr-AMP or anti-Thr-AMP antibodies on ATP synthase dimers, monomers, and subunits separated by two-dimensional electrophoresis revealed six bands. Based on the amount of these proteins, it is highly likely that the bands at approximately 55 kDa correspond to Atp1 and Atp2, while the 35 kDa band likely corresponds to Atp3. The interpretation of the remaining signals remains uncertain due to the limited resolution of smaller ATP synthase subunits in the gel. The signal derived exclusively from the dimers could potentially be attributed to Atp20 (g) or Atp21 (e). Although the antibodies used are well-established tools for AMPylation detection, the observed immuno-reactivity cannot be considered definitive evidence for AMPylation of individual ATP synthase subunits, but rather supports the presence of AMP-related modifications within ATP synthase-enriched fractions.

**Figure 4:**
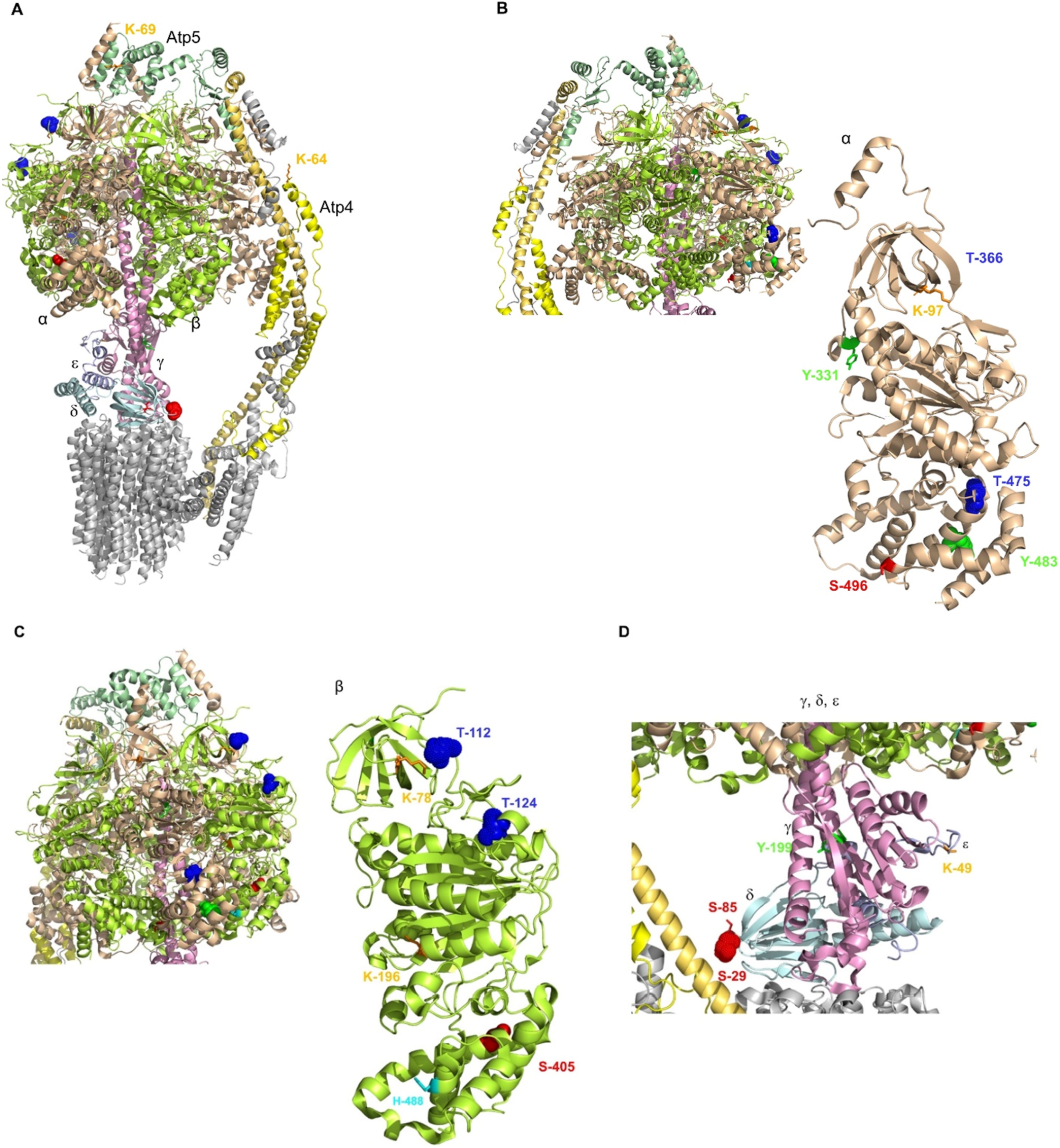
Distribution of AMPylated amino acid residues in the structure of ATP synthase subunits. (A) Overview of the ATP synthase monomer: An overview of the ATP synthase monomer, highlighting the distribution of AMPylated residues in the structure. (B) The F1 domain and modifications in subunit α: The F1 domain and modifications in subunit α are shown. AMPylated residues are displayed as sticks, with residues that are both phosphorylated and AMPylated indicated as spheres. (C) The F1 domain and modifications in subunit β: The F1 domain and modifications in subunit β are illustrated similarly, with AMPylated residues as sticks and phosphorylated-AMPylated residues as spheres. (D) The central stalk subunit modifications: Subunits in the central stalk and their modifications are shown. AMPylated residues are depicted as sticks, with dual-modified residues shown as spheres. Subunits and modified residues are color-coded as follows: Subunits: α—pale, β—gray, Atp5—pale green, Atp7—pale yellow, γ—light pink, δ—pale cyan.

We demonstrated the biological significance of the Atp16-S29 residue modification. Deletion of the *ATP16* gene leads to a significant destabilization of the mitochondrial genome, resulting in 100% of ρ^–^/ρ^0^ (*petites*) cells.^67,83^ Yeast cells lacking the Atp16 subunit cannot survive when synthesizing ATP synthase proton channel due to the loss of control over proton translocation by ATP synthesis, leading to proton slip.^68^ How the coupling of ATP synthase is controlled is still not fully understood, and post-translational modifications may be one of the mechanisms. To address this, Atp16 variants were expressed from plasmid-borne copies of the gene. Both Atp16 and Atp16-S29>A levels were slightly increased under these conditions, whereas Atp16-S29>E levels remained unchanged. This correlates with the elevated levels of endogenously expressed Atp16 in *fmp40*Δ cells, suggesting that Fmp40 plays a role in regulating Atp16 levels. The functionality of the Atp16 variants was confirmed by the complementation of the *petite* phenotype in the *atp16*Δ strain. Our data show that the AMPylation or phosphorylation of serine at position 29 in Atp16 is directly involved in regulating ATP synthase coupling—specifically controlling proton flow through the enzyme’s membrane domain, particularly under fermentative growth conditions. The loss of this regulatory control is compensated by ρ^–^/ρ^0^ mutations. Although we did not observe an increase in the population of *petites* cells when cultured on a nonfermentative carbon source, a subtle decrease in the inner membrane potential was detected. In contrast to cells lacking the Atp16 subunit, where the decrease in inner membrane potential is compensated by increased respiratory chain activity (very high oxygen consumption with NADH at state 4,^68^), this activity is reduced in *atp16-S29>A/E* cells. Thus, in these mutants, proton slip through the ATP synthase proton channel is not compensated by the increased oxygen consumption at state 4, indicating that the coupling between respiratory chain activity —responsible for creating the inner mitochondrial membrane potential in wild-type cells— and ATP synthase activity is lost. Our findings suggest that AMPylation or phosphorylation of Atp16 at serine 29 may represent one of the regulatory mechanisms controlling ATP synthase function and OXPHOS coupling. Nevertheless, the molecular consequences of this particular residue’s post-translational modifications, as well as within other ATP synthase subunits, remain largely unresolved and require further investigation. Ongoing studies in our laboratory are focused on determining whether AMPylation of ATP synthase residues is directly dependent on the Fmp40 AMPylase. Understanding the regulatory mechanisms of OXPHOS coupling is crucial, as ATP synthase dysfunction—manifested as H^+^ slip associated with ATP synthase rotor free-wheeling—has been shown to dissipate ΔΨ and contribute directly to mitochondrial disorders.^84–88^

## Study Limitations

Although this study used freshly isolated mitochondria that enables near-physiological AMPylation analysis, the isolated mitochondria may alter endogenous metabolic and redox states that can affect post-translational modifications. Furthermore, because AMPylation is typically substoichiometric, our shotgun proteomics approach without dedicated enrichment likely captured only the most abundant or stable modifications. The use of data-dependent acquisition (DDA) further biased peptide selection toward highly abundant precursor ions. This may underrepresent low-intensity AMPylated species. Consequently, suboptimal fragmentation spectra introduced uncertainty regarding precise PTM site localization, necessitating the tiered site probability thresholds (50–75%) applied in this study. While well-characterized antibodies provide strong supporting evidence for AMP-related modifications within ATP synthase-enriched fractions, they cannot unequivocally confirm site-specific AMPylation of individual subunits. Definitively resolving the mitochondrial AMPylome and validating specific targets will require integrating specific MS enrichment strategies with optimized collision energies alongside targeted mutagenesis, *in vitro* AMPylation assays, and biochemical discrimination from other nucleotide modifications.^89,90^

## Conclusion

Although AMPylation in bacteria has gained renewed interest in recent years, and *in vitro* and *in vivo* screens of AMPylated proteins in eukaryotes have been developed, our understanding of the biological processes regulated by this post-translational modification remains limited. The results presented here offer an initial perspective on the scope of AMPylation in yeast mitochondria. Our findings pave the way for further validation and substrate-targeted investigations that will help elucidate the specific consequences of AMPylation on substrate structure and function. Additionally, the fact that multiple amino acid residues within ATP synthase itself were identified as carrying both modifications—phosphorylation and AMPylation—indicates that the mitochondrial AMPylome should not be considered in isolation, but rather as part of a broader and highly interconnected post-translational regulatory network. Given that 135 out of the 274 *SELENOO* gene variants listed in the ClinVar database have been associated with diseases, research on SelO function will be instrumental in advancing our understanding of disease mechanisms.

## Supplementary Material













## Data Availability

The list of proteins and peptides identified in the mass spectrometry analysis is available in Supplementary Tables S2, S3, S4, and S5. The mass spectrometry proteomics data have been deposited to the ProteomeXchange Consortium (http://proteomecentral.proteomexchange.org) via the PRIDE^61^ partner repository with the data set identifier PXD058585 and 10.6019/PXD058585.

## Notes

Growth of Fmp40-deficient cells in liquid media and full-gel separation of ATP synthase complexes and subunits (Figure S1); examples of spectra from Atp2 and Atp16 are shown with the b-ions and y-ions used for identification marked (Figure S2); AMPylation was detected in ATP synthase purified from native mitochondria (Figure S3); representative images of wild-type, *atp16-S>A*, and *atp16-S>E* cells stained with MitoTracker Red CMXRos (Figure S4); list of AMPylation and phosphorylation sites identified within ATP synthase subunits (Table S6); influence of the *atp16* mutations on yeast mitochondrial respiration and ATP synthesis (Table S7); supplementary raw images of Figure 2; supplementary raw images of Figure S3A; supplementary raw images of Figure 3G; photographs of the full membranes corresponding to the experiments shown in Figure 2 (Figure S5); photographs of the full membranes corresponding to the experiments shown in Figure S3A (Figure S6); photographs of the full membranes corresponding to the experiments shown in Figure 3G (Figure S7) (PDF)
Yeast Mitochondrial Proteome (Tables S1) (XLSX)
Comprehensive details on the proteins found to carry post-translational modifications in each experiment (Tables S2) (XLSX)
List of identified peptides and amino acid residues carrying AMP modifications (Tables S3) (XLSX)
Lists of phosphorylated peptides identified in all data sets (Tables S4) (XLSX)
All identified phospho-sites and phosphoproteins, along with the results of the comparison with the YeastMine database (Tables S5) (XLSX)

## Abbreviations

AMP: adenosine monophosphate
BN-PAGE: bleu native polyacrylamide gel electrophoresis
BSA: bovine serum albumin
CCCP: carbonyl cyanide m-chlorophenylhydrazone
ΔΨ: mitochondrial inner membrane potential
FAIMS: an atmospheric pressure ion mobility MS technique
MS: mass spectrometry
mtDNA: mitochondrial DNA
OD: optical density
OXPHOS: oxidative phosphorylation
PTM: posttranslational modification
ρ+: containing mtDNA
ρ^–^/ρ^0^: partial/total deletion of mtDNA
ROS: reactive oxygen species
SDS-PAGE: denaturing polyacrylamide gel electrophoresis
SGD: *Saccharomyces* Genome Database
SelO: Selenoprotein O
TMPD: *N*,*N*,*N*′,*N*′-Tetramethyl-*p*-phenylenediamine
TCA: tricarboxylic acid cycle
