# Supplementary material for: Profiling of Yeast Saccharomyces cerevisiae Mitochondrial AMPylome Reveals a Regulation of OXPHOS Coupling through ATP Synthase Subunit Delta
Source: J Proteome Res. 2026 Jul 28;25(8):3965–86. doi: 10.1021/acs.jproteome.5c01273 (PMC13459536; doi:10.1021/acs.jproteome.5c01273)
Supplement: Supplementary file 1 [file pr5c01273_si_001.pdf]

Supporting Information

Profiling of yeast *Saccharomyces cerevisiae* mitochondrial AMPylome reveals a regulation of OXPHOS coupling through ATP synthase subunit delta

Chiranjit Panja, Aneta Wiesyk, Suchismita Masanta, Katarzyna Niedzwiecka, Emilia Baranowska, Dominik Cysewski, Marta Sipko, Anna Anielska-Mazur, Agata Malinowska, Roza Kucharczyk*
Institute of Biochemistry and Biophysics, Polish Academy of Sciences, Pawinskiego 5A, 02-106 Warsaw, Poland
*Corresponding authors: roza@ibb.waw.pl

List of contents:

1. Figure S1. Growth of Fmp40-deficient cells in liquid media and full-gel separation of ATP synthase complexes and subunits ……………………………………………………………..p. S2
2. Figure S2. Examples of spectra from Atp2 and Atp16 are shown with the b-ions and y-ions used for identification marked. ……………………………………………………………p. S3
3. Figure S3. The AMPylation was detected in ATP synthase purified from native mitochondria…………………………………………………………………………………p.S4
4. Figure S4. The representative images of wild-type, *atp16-S>A,* and *atp16-S>E* cells stained with MitoTracker Red CMXRos. ………………………………………………………….p. S5
5. Table S6. List of AMPylation and phosphorylation sites identified within ATP synthase subunits.………………………………………………………………..……………………p.S6
6. Table S7. Influence of the atp16 mutations on yeast mitochondrial respiration and ATP synthesis.…………………………………………………………………………………...…p.S10
7. Figure S5. Photographs of the full membranes corresponding to the experiments shown in Fig. 2…………………………………………………………………..…………………p.S11
8. Figure S6. Photographs of the full membranes corresponding to the experiments shown in Fig. S3A.…………………………………………...…………….…………………..p.S12
9. Figure S7. Photographs of the full membranes corresponding to the experiments shown in Fig. 3G. ……………………………..…………………………………..………………p.S13
10. Supplementary Table S1 – Yeast Mitochondrial Proteome (XLSX).
11. Supplementary Table S2 - Comprehensive details on the proteins found to carry post-translational modifications in each experiment (XLSX).
12. Supplementary Table S3 - List of identified peptides and amino acid residues carrying AMP modifications (XLSX).
13. Supplementary Table S4 - Lists of phosphorylated peptides identified in all datasets (XLSX).
14. Supplementary Table S5 - All identified phospho-sites and phosphoproteins, along with the results of the comparison with the YeastMine database (XLSX).

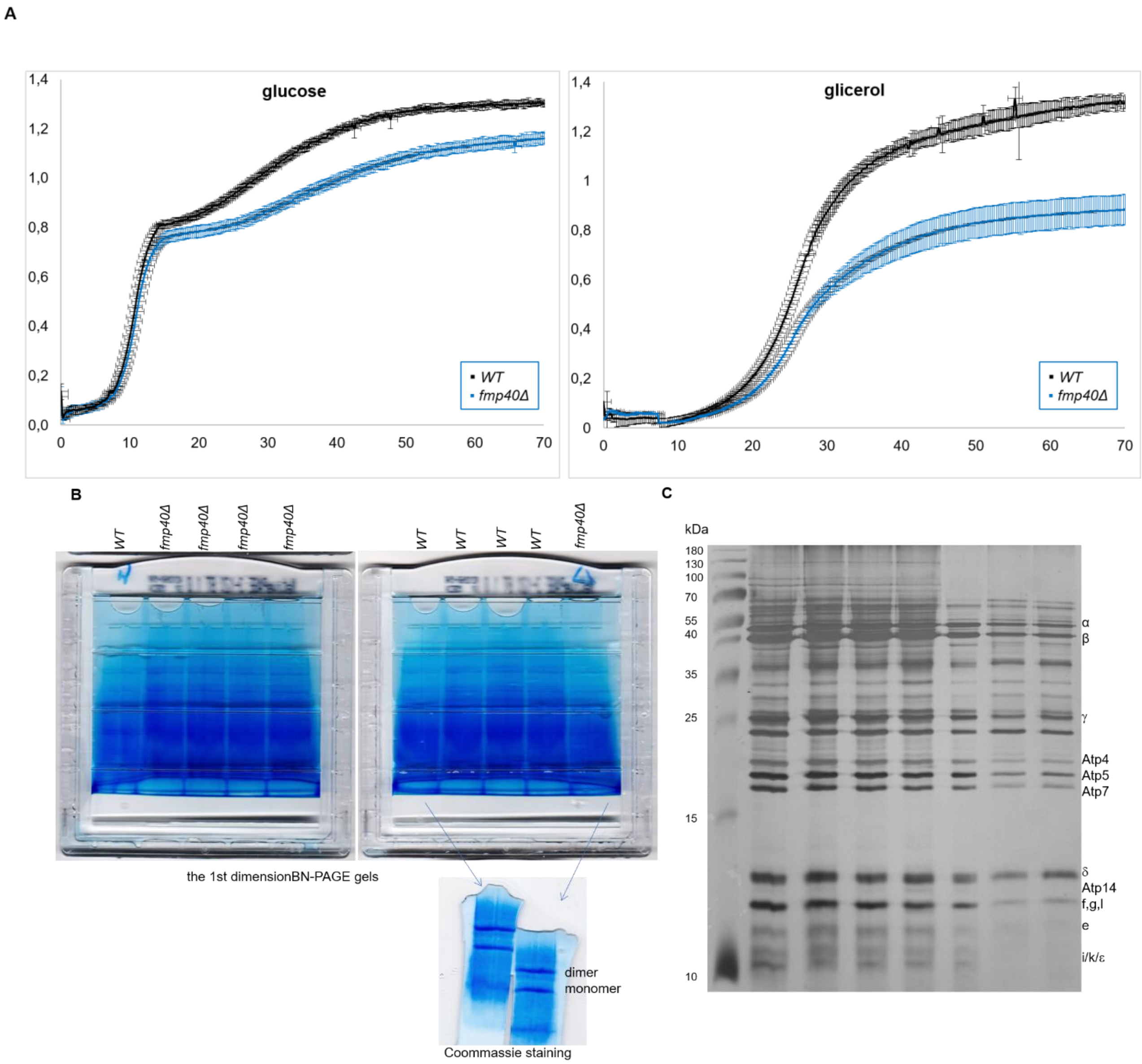

Figure S1. Growth of Fmp40-deficient cells in liquid media and full-gel separation of ATP synthase complexes and subunits. **A)** Growth of wild-type yeast cells and *fmp40Δ* mutant cells in a rich liquid medium containing glucose or glycerol as a sole carbon source at elevated temperature for yeast cells – 36 °C. The cultures were inoculated with cells freshly grown in glucose and monitored with a Bioscreen CTM system. **B)** The 1st dimension BN-PAGE gels separating the dimers and monomers of ATP synthase were used for the 2nd dimension SDS-PAGE or mass spectrometry analysis. The ATP synthase dimers and the monomers were extracted from 1 mg of the mitochondria with 1.5 % digitonin and run on NativePAGE™ 3–12 % Bis-Tris Gels. Gels were photographed before further processing. The indicated lanes were stained with Coomassie stain. **C)** Silver stained gel separating the ATP synthase subunits after extraction of its complexes from the mitochondrial membrane and pull down by Atp6-HA-6xHis. The eluates were separated in 15 % SDS-PAGE and the bands were visualized by silver staining. The dominating proteins identified in bands by mass spectrometry are indicated on the right. The representative silver stained gel is shown. Each lane contains an independent biological repeat from the same experiment.

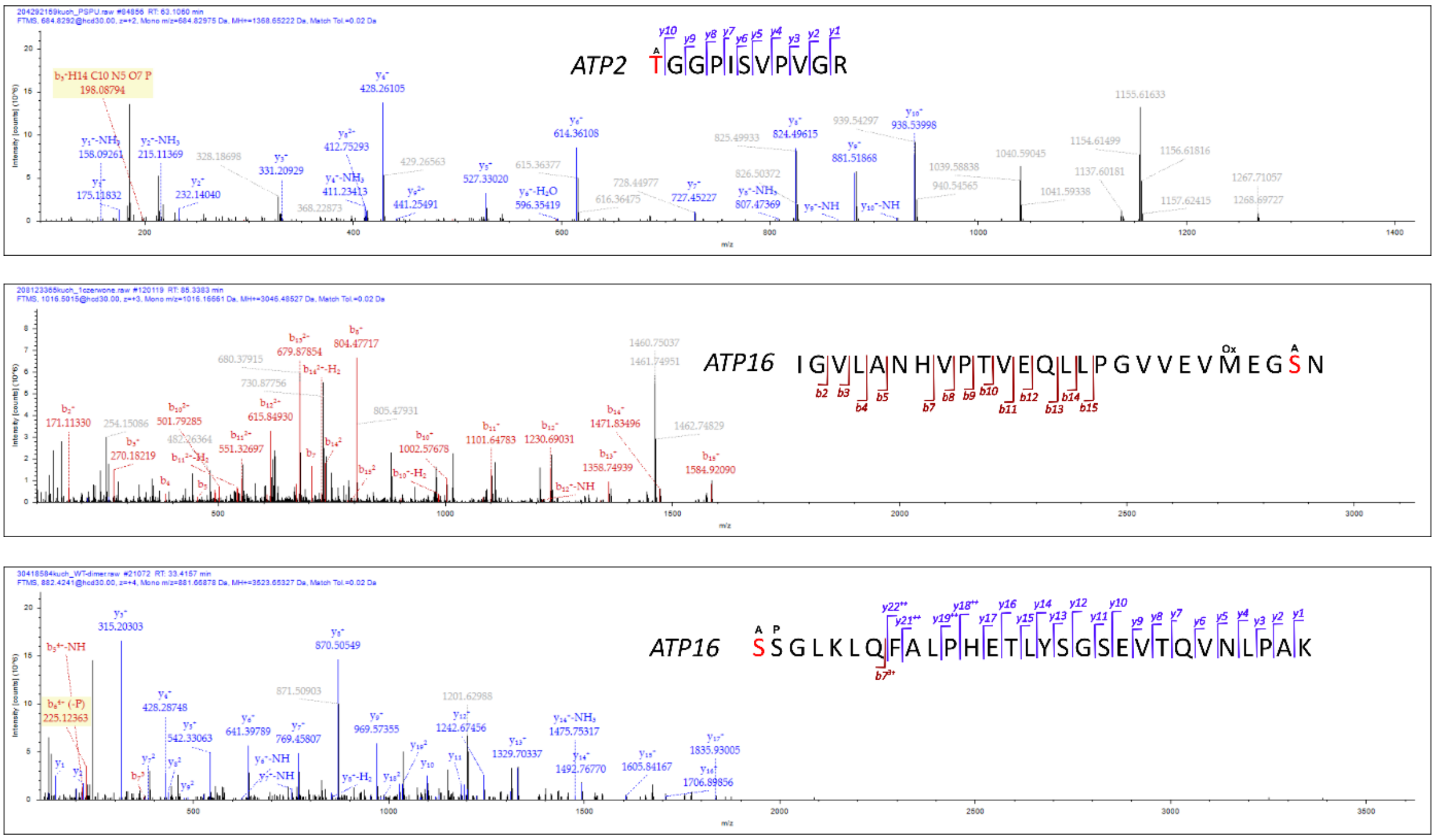

Figure S2. Examples of spectra from Atp2 and Atp16 are shown with the b-ions and y-ions used for identification marked. The post-translational modifications are highlighted in red. In some cases, the ion carrying AMP is not annotated; peptide identification and AMP site localization are performed with probabilistic algorithms implemented in Proteome Discoverer (SEQUEST, Percolator, IMP-ptmRS), using high-precision parent-ion mass measurements and existing MS/MS data within the yeast protein sequence database.

.

A

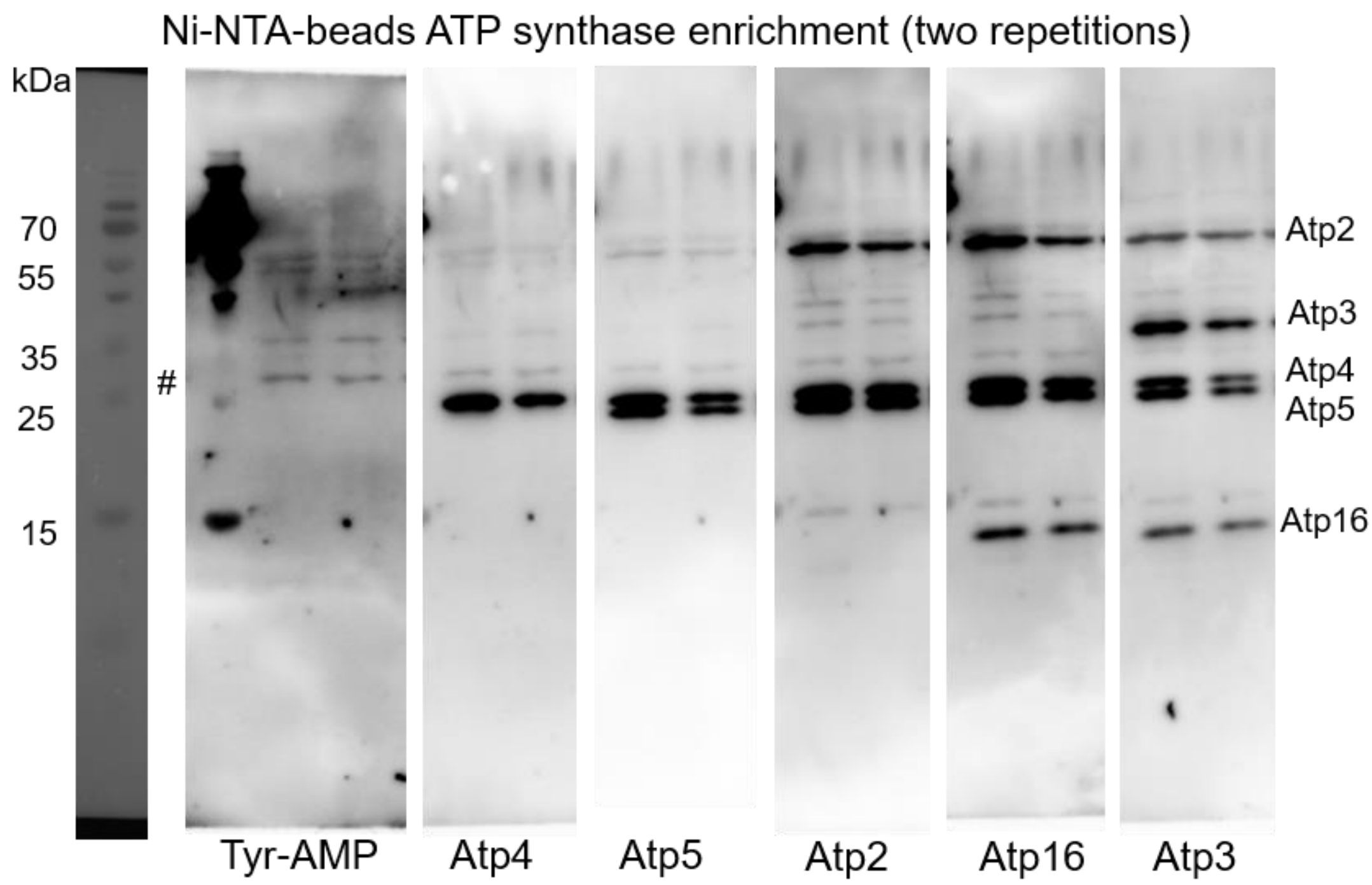

B

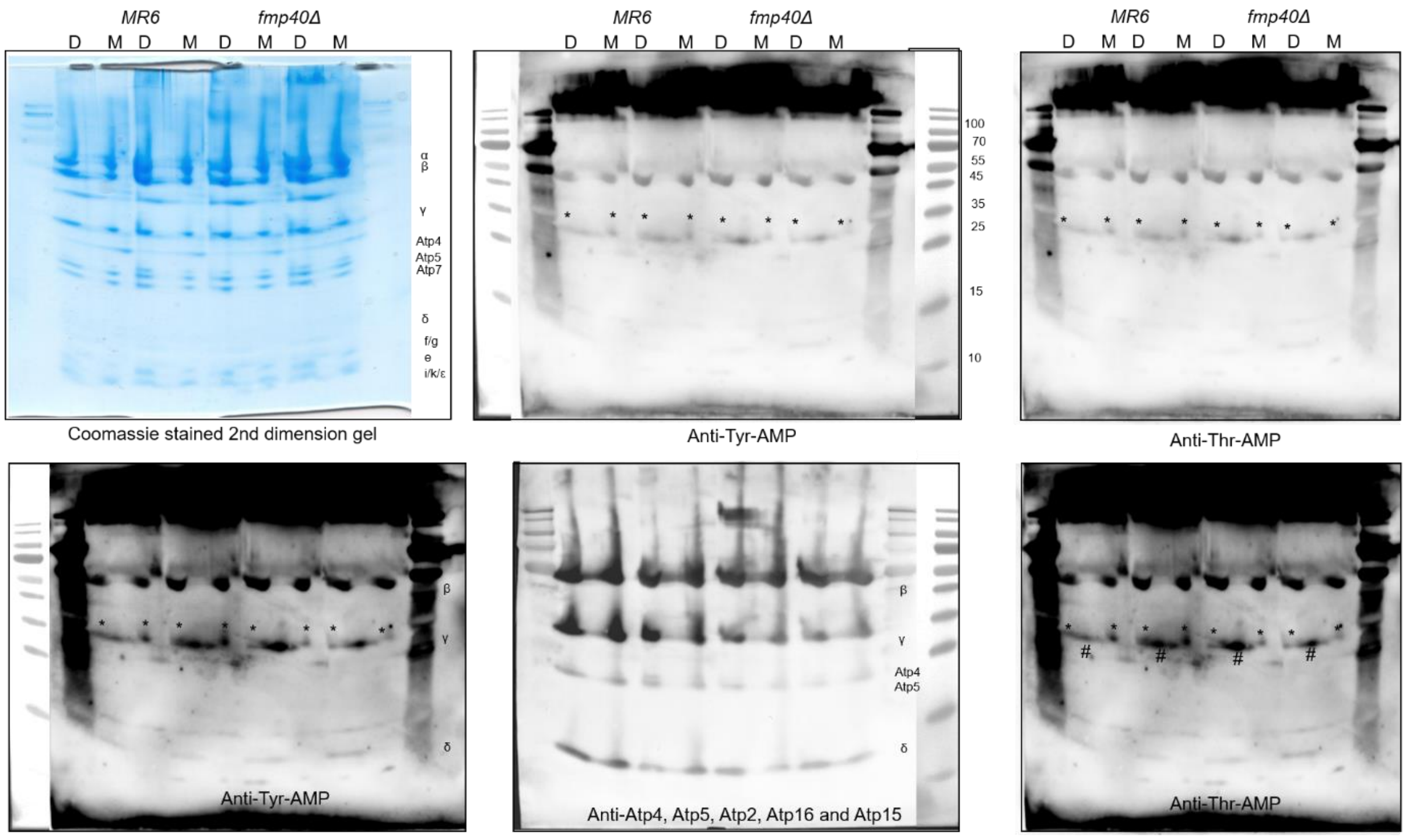

Figure S3. The AMPylation was detected in ATP synthase purified from native mitochondria. **A)** The whole ATP synthase complexes were purified from mitochondria of a wild-type strain expressing subunit Atp6-HA-6xHis by Ni-NTA agarose beads, and 2 µg of Ni-NTA bead eluates were loaded on the 15 % SDS-PAGE gel. After transfer, the membrane was incubated sequentially, without stripping, with the following antibodies against: AMP-Tyr (ref. ABS184, Merck), Atp4, Atp5, Atp2, Atp16, and Atp3. Each lane contains an independent biological repeat; five repetitions were done, and two are shown. # - unknown protein, no ATP synthase

subunit. Original blots are presented in Fig. S6, page S12. The corresponding silver-stained gel is shown in Fig. S1B, S1C. **B)** The 2nd dimension gel after separation of ATP synthase dimers (D) and monomers (M), solubilized from the native mitochondria of wild-type and *fmp40Δ* cells, were stained by Coomassie after the transfer of proteins to the membrane. The membrane was incubated with anti-Tyr-AMP (ref. ABS184, Merck), dehybridized, then with anti-Thr-AMP (ref. 09-890, Merck), dehybridized again, and incubated subsequently with anti-ATP synthase subunits Atp2, Atp4, Atp5, Atp16, and Atp3. # - not identified protein from CIII or CIV, not ATP synthase subunit. * AMP signal seems to be absent / reduced in *fmp40Δ*.

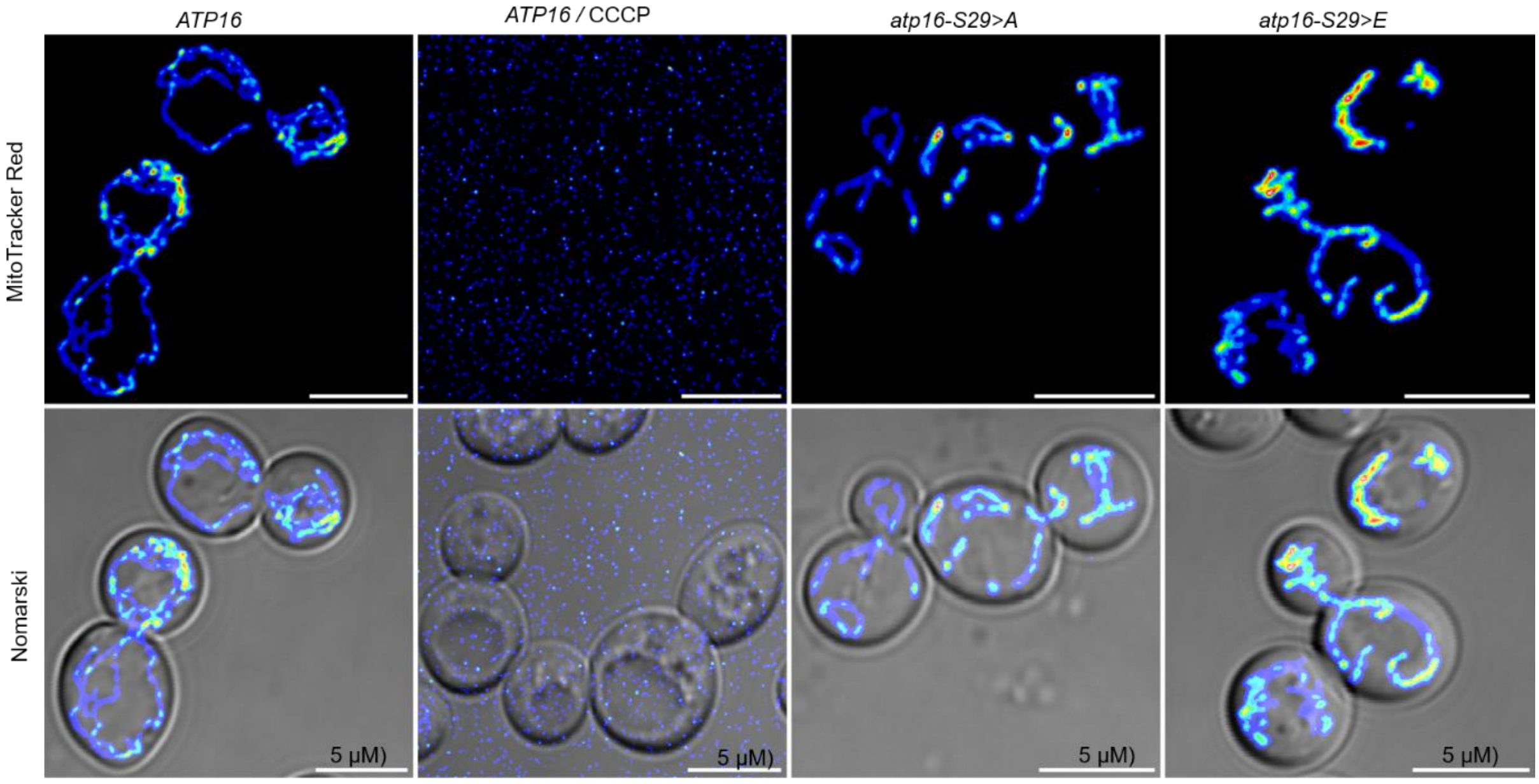

Figure S4. The representative images of wild-type, *atp16-S>A,* and *atp16-S>E* cells stained with MitoTracker Red CMXRos. The CCCP-treated cells were introduced as a negative control. The four biological repetitions from each strain were done. The scale bar represents 5 µm. Related to Fig. 3F.

Table S6. Modifications identified within ATP synthase subunits.

Subunit (published P-sites)**	Identified AMP-peptide	Identified Phospho-peptide	Position of the AMP / P amino acid[b], ***
Atp1/α		[K].AQPTEVSSILEERIK.[G] / [K].AQPTEVSSILEER.[I]	T-43
(S-37,		[R].IKGVSDEANLNETGR.[V] / [K].GVSDEANLNETGR.[V]	S-57
T-43,		[V].FGLNNIQAEELVEFSSGVKGMALNLEPGQVGIVLFGSDR.[L]	S-93
T-38,		[V].FGLNNIQAEELVEFSSGVKGMALNLEPGQVGIVLFGSDR.[L]	S-94
S-46,	[N].IQAEELVEFSSGVKGMALNLEPGQVGIVLFGSDR.[L]		K-97
S-47,		[E].LVKRTGNIVDVPVGPGLLGR.[V] / [R].TGNIVDVPVGPGLLGR.[V]	T-128
S-57,		[R].RSVHEPVQTGLK.[A] / [R].RSVHEPVQTGLK.[A] / [R].RSVHEPVQTGLK.[A]	S-178
T-128,		[K].TAVALDTILNQKR.[W] / [K].TAVALDTILNQK.[R]	T-213
S-178,		[K].LYCVYVAVGQK.[R]	Y-237
T-213		[K].LYCVYVAVGQK.[R]	Y-240
S-230,		[R].STVAQLVQTLEQHDAMK.[Y] / [R].STVAQLVQTLEQHDAMK.[Y]	S-248, T-249
S-233		[R].STVAQLVQTLEQHDAMK.[Y]	T-256
T-256		[F].TAASIGEWFR.[D]	T-286, T-289
S-309		[K].HALIVYDDLSK.[Q] / [K].HALIVYDDLSK.[Q]	Y-305
S-340	[L].SLLLRRPPGREAYPGDVFYLHSR.[L]*		Y-331
S-350,		[R].EAYPGDVFYLHSR.[L] / [L].SLLLRRPPGREAYPGDVFYLHSR.[L]	Y-331
S-413,	[K].LSEKEGSGSLTALPVIETQGGDVSAY.[I]		T-366
S-445,	[Q].YSPLATEEQVPLIYAGVNGHLDGIELSR.[I]	[E].RLTQLLKQNQYSPLATEEQVPLIYAGVNGHLDGIELSR.[I]	T-462, T-475
S-507,		[Q].NQYSPLATEEQVPLIYAGVNGHLDGIELSR.[I]	Y-470, T-475
S-511,		[Q].YSPLATEEQVPLIYAGVNGHLDGIELSR.[I]	S-471
S-527,	[P].LIYAGVNGHLDGIELSR.[I] / [S].PLATEEQVPLIYAGVNGHLDGIELSR.[I]*	[Q].NQYSPLATEEQVPLIYAGVNGHLDGIELSR.[I]	Y-483
S-533)	[E].LSRIGEFESSFLSYLK.[S]		S-496
		[K].SNHNELLTEIR.[E]	S-511
Atp2/β	[R].CMASAAQSTPITGK.[V]*	[M].ASAAQSTPITGK.[V]	S-35
(S-35,		[G].AIVDVHFEQSELPAILNALEIKTPQGKLVLEVAQHLGENTVR.[T]	S-61, T-74
S-39,	[K].TPQGKLVLEVAQHLGENTVR.[T]*		K-78
T-40,		[R].TIAMDGTEGLVRGEK.[V]	T-94
T-43		[T].IAMDGTEGLVRGEKVLDTGGPISVPVGR	T-100
T-112,	[D].TGGPISVPVGR.[E] / [D].TGGPISVPVGR.[E]*	VLDTGGPISVPVGR.[E] [K]. / [K].VLDTGGPISVPVGRETLGR.[I]	T-112
S-117,	[R].ETLGRIINVIGEPIDERGPIK.[S]		T-124
T-124,		[R].KPIHADPPSFAEQSTSAEILETGIK.[V]	S-156
T-237,		[A].DPPSFAEQSTSAEILETGIK.[V] / [K].LRKPIHADPPSFAEQSTSAEILETGIK.[V]	S-161,T-162,S-163
S-246,	[A].GVGKTVFIQELINNIAK.[A]*		K-196

S-299, S-302 T-317, S-373, T-470	[V].ASKVQETLQTYK.[S]<br>[N].IPEHAFYMVGGIEDVVAK.[A]	[A].GVGKTVFIQELINNIAK.[A] / [L].FGGAGVGKTVFIQELINNIAK.[A]<br>[K].AHGGFSVFTGVGER.[T]<br>[R].EMKETGVINLEGESK.[V] / [K].ETGVINLEGESK.[V]<br>[V].ALTGLTIAEYFR.[D]<br>[R].FTQAGSEVSALLGR.[I]<br>[R].IPSAVGYQPTLATDMGLLQER.[I] / Q].PTLATDMGLLQER.[I]<br>[G].SVTSVQAVYVPADDLTDPAPATTFAHLDATTVLSR.[G] / [<br>[K].KGSVTSVQAVYVPADDLTDPAPATTFAHLDATTVLSR.[G]<br>[G].SVTSVQAVYVPADDLTDPAPATTFAHLDATTVLSR.[G]<br>[G].SVTSVQAVYVPADDLTDPAPATTFAHLDATTVLSR.[G]/[P].ADDLTDPAPATTFAHLDATTVLSR.[G]<br>[P].APATTFAHLDATTVLSR.[G] / [D].PAPATTFAHLDATTVLSR.[G]<br>[D].ATTVLSRGISELGIYPAVDPLDSK.[S]<br>[R].GISELGIYPAVDPLDSK.[S] / [I].SELGIYPAVDPLDSK.[S]<br>[P].LDSKSRLLDAAVVGQEHYDVASK.[V]<br>[R].LLDAAVVGQEHYDVASK.[V] / [R].LLDAAVVGQEHYDVASK.[V]/<br>[A].VLEGKYDNIPEHAFYMVGGIEDVVAK.[A] / [N].IPEHAFYMVGGIEDVVAK.[A /<br>[A].VLEGKYDNIPEHAFYMVGGIEDVVAK.[A] / [N].IPEHAFYMVGGIEDVVAK.[A] /<br>[K].YDNIPEHAFYMVGGIEDVVAK.[A] / [H].AFYMVGGIEDVVAK.[A] /	T-197<br>S-215<br>T-237<br>T-268<br>T-295<br>S-310, Y-314, T-317<br>S-336,<br>T-338<br>Y-344<br>T-351<br>T-357, T-358<br>S-369, S-373,<br>S-373, Y-378<br>S-386, S-388<br>Y-401, S-405<br>Y-482, Y-491<br><br><br>H-488
Atp3/γ (S-7, S-65, S-85, T-98, T-100 S-122, T-142 S-176, S-185, S-224, S-226 S-226)	[K].ISIFYNDPVSSLSFEPSEKPIFNAK.[T]*	[K].ISAKKMDEAEQLFYK.[N]<br>[K].NAETKNLDVEATETGAPK.[E] / [K].NAETKNLDVEATETGAPK.[E]<br>[K].NLDVEATETGAPK.[E]<br>[I].VAITSDKGLCGSIHSQLAK.[A]<br>[K].GLCGSIHSQLAK.[A]<br>[R].HLNDQPNADIVTIGDK.[I] / [R].RHLNDQPNADIVTIGDK.[I]<br>[L].SINGIGKDAPTFQESALIADK.[L]<br>[K].DAPTFQESALIADK.[L]<br><br>[D].PVSSLSFEPSEKPIFNAK.[T]<br>[L].SFEPSEKPIFNAK.[T]<br>[K].TIEQSPSFGK.[F]<br>[K].TIEQSPSFGKFEIDTDANVPR.[D]<br>[K].FEIDTDANVPR.[D]	S-73<br>T-90<br>T-98<br>T-111<br>S-119<br>T-142<br>S-162<br>T-172<br>Y-199<br>S-205<br>S-207<br>T-220<br>S-224, S-226<br>T-234
Atp4/b (T-39 S124, S144, S-146 S-162		[Y].MSSTPEKQTDPK.[A] / [M].SSTPEKQTDPK.[A]<br>[K].ANSIINAIPGNNILTK.[T]<br>[K].AKANSIINAIPGNNILTKTGVLGTSA.[A]<br>[K].YLAPAYKDFADAR.[M]<br>[K].KVSDVLNASR.[N]<br>[R].IDSVSQLQNVAETTK.[V] / [K].DRIDSVSQLQNVAETTK.[V]	S-38<br>S-52<br>T-64, T-66, T-71<br>Y-107<br>S-124<br>S-144, S-146

S-217)		[K].DRIDSVSQLQNVAETTK.[V]<br>[K].ETVELESEAFELK.[Q]<br>A].KSVISRVQSELGNPK.[F]	T-155<br>T-165<br>S-213
Atp5 /OSCP (S-48, S-62, S-86, T-139, S-161 S-196, T-199)	[N].PKLGHLLLNPALSLKDR.[N]*	[A].AAPPPVRLFGVEGTYATALYQAAAK.[N] / [A].PPPVRLFGVEGTYATALYQAAAK.[N]<br>[T].YATALYQAAAK.[N] / [T].YATALYQAAAK.[N]<br>[K].NSSIDAAFQSLQK.[V]<br><br>[K].LGHLLLNPALSLKDR.[N]<br>[K].DRNSVIDAIVETHK.[N] / [R].NSVIDAIVETHK.[N]<br>[C].FEKIASDFGVLNDAHNGLLK.[G] / [E].KIASDFGVLNDAHNGLLK.[G] /<br>[K].IASDFGVLNDAHNGLLKG.[T] / [E].KIASDFGVLNDAHNGLLK.[G]<br>[K].GTVTSAEPLDPK.[S]<br>[K].SLKLENVVKPEIK.[G]	T-35<br>Y-36, T-38<br>S-48, S-49<br>K-69<br>S-80<br>S-86, T-94<br><br>S-123<br><br>T-139<br>S-169
Atp7/d (T-23 S-25, S-31, S-32, S-67, S-95 S-109, S-112, S-120, T-121, S-127)	[S].VLKNTSVIDKIESYV.[Q]	[K].VISSLRITGSTATQLSSFK.[K] / [R].ITGSTATQLSSFK.[K]<br>[R].ITGSTATQLSSFKK.[R]<br>[R].QLLELQSQPTEVDFSHYRSVLK.[N]<br><br>[S].VLKNTSVIDKIESYVK.[Q] / [K].NTSVIDKIESYVK.[Q]<br>[K].ETESLVSK.[E]<br>[K].DLQSTLDNIQSARPFDELTVDDLTK.[I] / [K].DLQSTLDNIQSAR.[P]<br>[K].DLQSTLDNIQSARPFDELTVDDLTK.[I]<br>[R].PFDELTVDDLTK.[I]	S-19, T-23<br>S-25<br>S-49<br>K-64<br>T-66, S-67<br>T-107<br>S-120, T-121<br>S-127<br>T-135
Atp15/ε (T-32, S-34, S-39, T-41, T46, T-52)	[Y].KNGTAASEPTPITK.[-]*	[S].AWRKAGISYAAYLNVAAQAIR.[S]<br>[R].SSLKTELQTASVLNR.[S]<br>[K].TELQTASVLNR.[S]<br>[R].SQTDAFYTQYK.[N]<br><br>[K].NGTAASEPTPITK.[-]<br>[I].VAITSDKGLCGSIHSQLAK.[A]<br>[L].SINGIGKDAPTFQESALIADK.[L]	S-10<br>S-24, S-25<br>T-28, T-32<br>S-39, T-41<br>K-49<br>T-52<br>T-111<br>S-162, T-172
Atp16/δ (S-21, S-29, S-30, S-45, S-47, S-135, S-136,	[A].SSGLKLQFALPHETLYSGSEVTQVNLPAK.[S]<br><br>[R].IGVLANHVPTVEQLLPGVVEVMEGSN.[S]*	[A].SSGLKLQFALPHETLYSGSEVTQVNLPAK.[S]<br>[A].SSGLKLQFALPHETLYSGSEVTQVNLPAK.[S]	S-29<br>S-30<br>S-85

S-137)			
Atp14/h (T-92, T-120)		[K].AYTEQNVETAHVAK.[E]*	T-86
Atp17/f S-17, S-18 S-23, S-39)		[K].SLPQGPAPAIK.[A]	S-39
Atp18/i NONE		[K].AADLSSNTKEFINDPR.[N]	S-34
Atp19/k NONE		[K].TVDIKTDNKDEEK.[F]	T-41
Atp20/g (S-3, S-62)		[K].QSLNFALKPTEVLSCLK.[N]	S-62, T-70
Atp21/e S-90		[A].KLHPVVTPKDVPANASFNLEDPNIDFER.[V]	T-61
Mco10/l (S-45, S-46, S-49, S-53, S-56, S-57, S-59, S-71)		[K].SGESSNSDAMGKDDDVVK.[S]<br>[K].SIEGFLNDLEKDTR.[Q]	S-53,S-56<br>S-71

a Phosphorylated / AMPylated amino acid is indicated by blue / red color, respectively. Residues where AMPylation and phosphorylation were identified are marked in both colors.

b Positions were determined from precursor protein sequences.

Peptide with modification present in ATP synthase pulldown with the Atp6-HA-6xHis from the wild type strain

Peptide with modification present ATP synthase complexes extracted from native gels

Peptide with modification present in percol purified mitochondria

Peptide with modification present in Fmp40-Myc pulldown Peptides highlighted in several colors were found in the experiments marked with these colors.

*- present in *fmp40Δ*

** – not found in our conditions

*** – new phosphorylation site found

Table S7. Influence of the *atp16* mutations on yeast mitochondrial respiration and ATP synthesis.

Mitochondria were isolated from wild-type and mutant strains grown for 5-6 generations in SC-glycerol-ura at 28°C. Reaction mixes for assays contained 0.075 mg/ml of protein, 4 mM NADH, 150 (for respiration assays) or 750 (for ATP synthesis) µM ADP, 12.5 mM ascorbate (*Asc*), 1.4 mM N,N,N,N,-tetramethyl-p-phenylenediamine (*TMPD*), and 4 µM CCCP. All cultures contained 100% of ρ^+ cells. The values reported are averages of triplicate assays ± standard deviation. Respiratory and ATP synthesis activities were measured using freshly isolated, osmotically protected mitochondria buffered at pH 6.8. * p value < 0.05.

Strain	Respiration rates, nmol O.min^{-1}.mg^{-1}				ATP synthesis rate, nmol ATP/min^{-1}/mg^{-1}	P/O
	NADH	NADH +ADP	NADH +CCCP	Asc/TMPD + CCCP		
WT	283±24	779±59	1252±75	2381±263	928±63	1,19±0,01
atp16-S>A	256±13	622±25*	854±8*	1703±75*	920±71	1,48±0,05*
atp16-S>E	228±29	546±18*	826±67*	1663±94*	838±62	1,53±0,06*

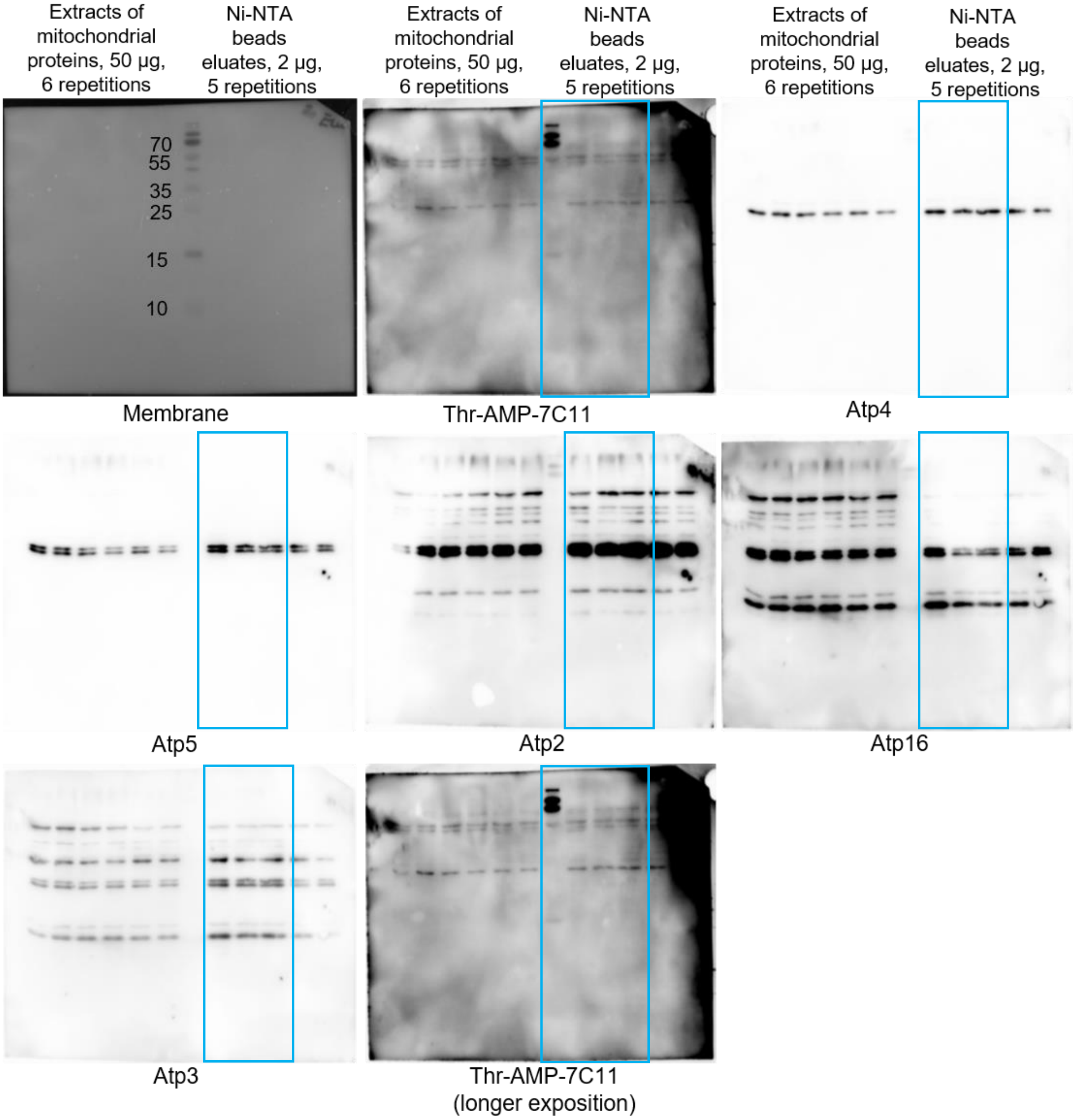

Figure S5. Photographs of the full membranes corresponding to the experiments shown in Fig. 2. The regions corresponding to the lanes presented in Fig. 2 are outlined with blue rectangles.

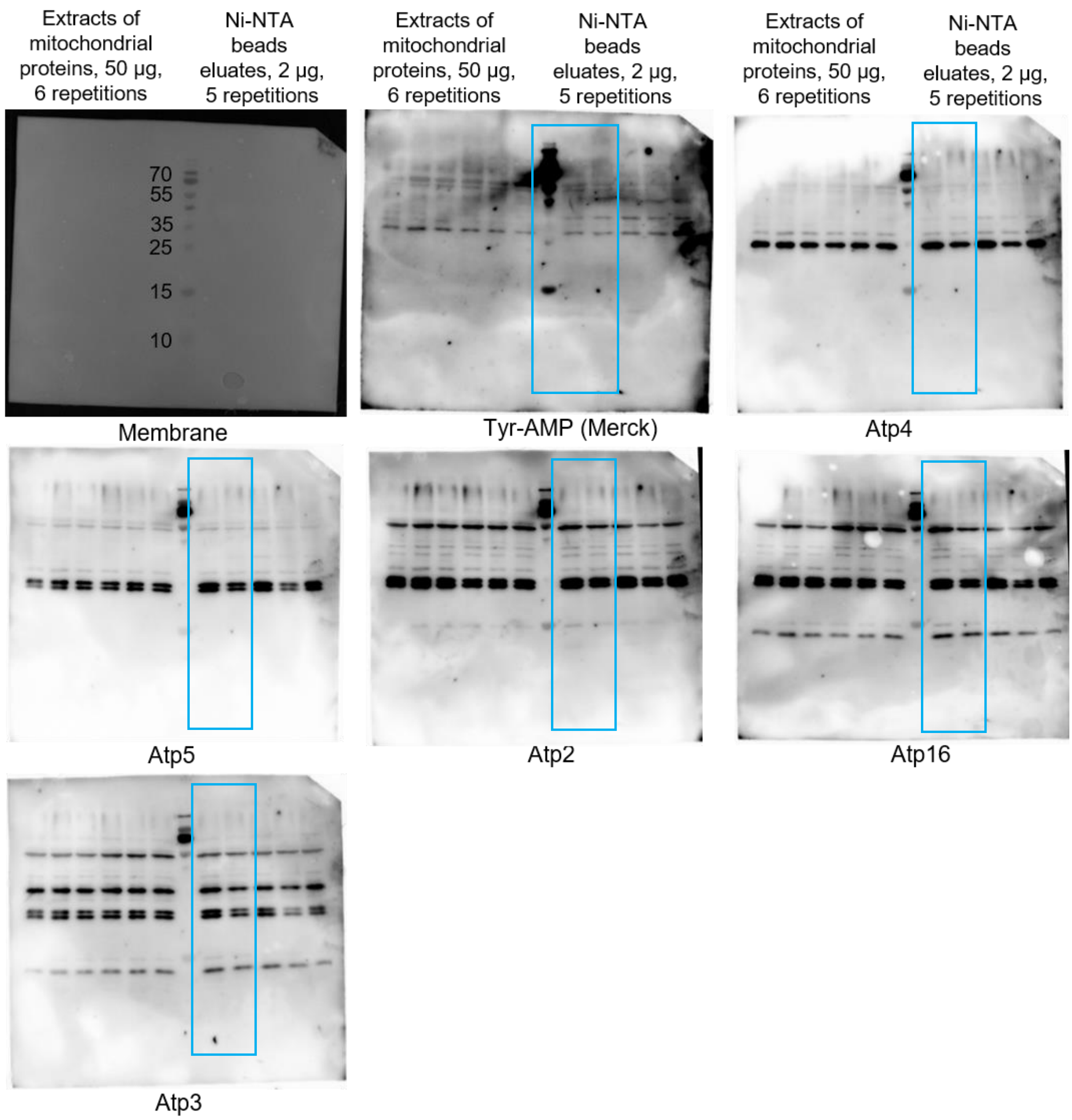

Figure S6. Photographs of the full membranes corresponding to the experiments shown in Fig. S3A. The regions corresponding to the lanes presented in Fig. S3A are outlined with blue rectangles.

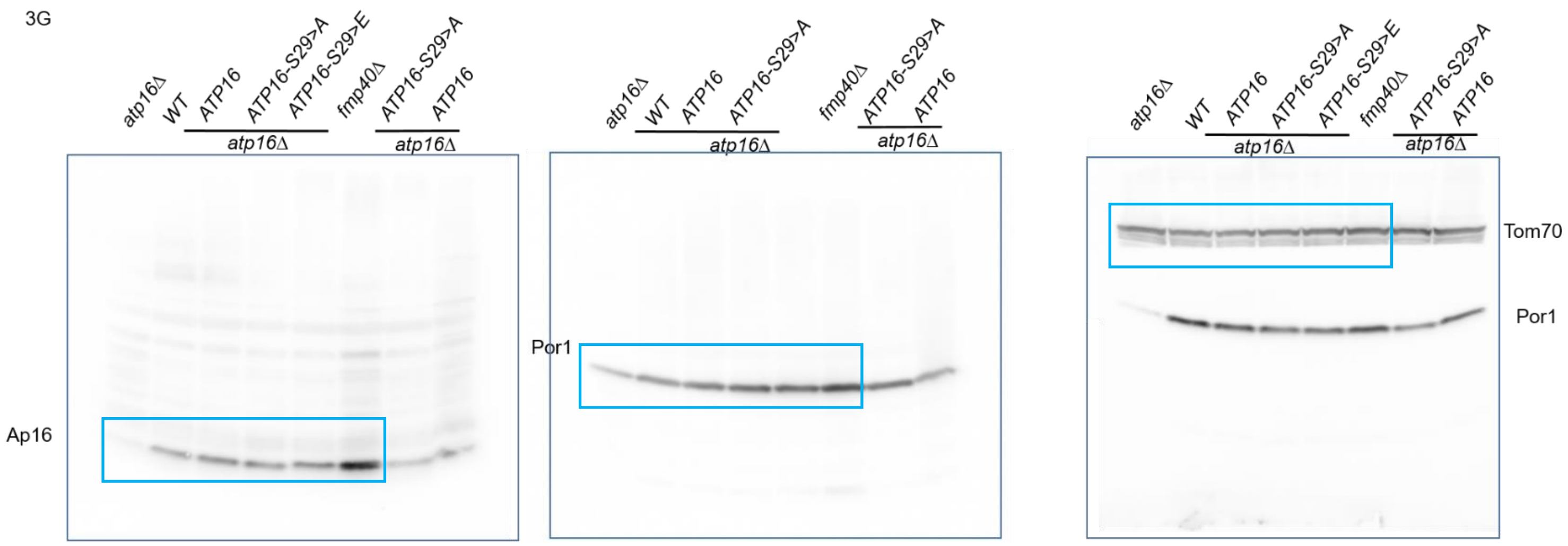

Figure S7. Photographs of the full membranes corresponding to the experiments shown in Fig. 3G. The regions corresponding to the lanes presented in Fig. 3G are outlined with blue rectangles.
